# RTN1 mediates progression of kidney disease by inducing ER stress

**DOI:** 10.1038/ncomms8841

**Published:** 2015-07-31

**Authors:** Ying Fan, Wenzhen Xiao, Zhengzhe Li, Xuezhu Li, Peter Y. Chuang, Belinda Jim, Weijia Zhang, Chengguo Wei, Niansong Wang, Weiping Jia, Huabao Xiong, Kyung Lee, John C. He

**Affiliations:** 1 Division of Nephrology, Department of Medicine, Icahn School of Medicine at Mount Sinai, New York 10029, USA; 2 Department of Nephrology, Shanghai Jiao Tong University Affiliated Sixth People's Hospital, Shanghai 200233, China; 3 Division of Nephrology, Department of Medicine, Jacobi Medical Center, Albert Einstein College of Medicine, Bronx, New York 10461, USA; 4 Immunology Institute, Department of Medicine, Icahn School of Medicine at Mount Sinai, New York, 10029, USA

## Abstract

Identification of new biomarkers and drug targets for chronic kidney disease (CKD) is required for the development of more effective therapy. Here we report an association between expression of reticulon 1 (RTN1) and severity of CKD. An isoform-specific increase in the expression of RTN1A is detected in the diseased kidneys from mice and humans, and correlates inversely with renal function in patients with diabetic nephropathy. RTN1 overexpression in renal cells induces ER stress and apoptosis, whereas RTN1 knockdown attenuates tunicamycin-induced and hyperglycaemia-induced ER stress and apoptosis. RTN1A interacts with PERK through its N-terminal and C-terminal domains, and mutation of these domains prevents this effect on ER stress. Knockdown of *Rtn1a* expression *in vivo* attenuates ER stress and renal fibrosis in mice with unilateral ureteral obstruction, and also attenuates ER stress, proteinuria, glomerular hypertrophy and mesangial expansion in diabetic mice. Together, these data indicate that RTN1A contributes to progression of kidney disease by inducing ER stress.

## Abstract (web summary)

ER stress is associated with the pathogenesis of chronic kidney disease (CKD) and new CKD therapies are needed. Here the authors show that expression of Rtn1 can control severity of renal disease and that inhibition of its expression can attenuate ER stress and CKD.

## Graphical abstract

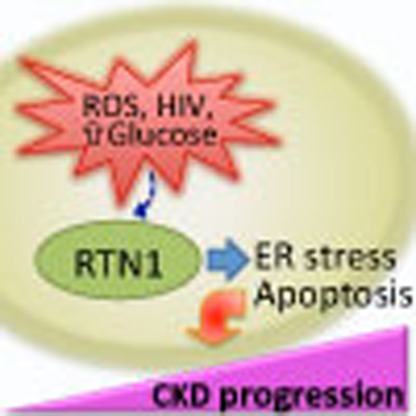

Chronic kidney disease (CKD) affects ∼10% of US adults. The incidence and prevalence of this condition are increasing worldwide^1^. Therapeutic options for CKD are limited and at best only offer partial protection against disease progression^2^,^3^,^4^. Therefore, there is an urgent need to identify key mediators of CKD progression for the development of more effective therapy to halt its progression.

In this study, to identify such potential mediators, we profiled the renal gene expression of a murine model of HIV-associated nephropathy (HIVAN), which has progressive CKD. Genes whose level of expression correlated with severity of renal injury were identified and among them was *Rtn1*, which encodes for an endoplasmic reticulum (ER)-associated protein called reticulon 1 (RTN1). The reticulon family has four members: *RTN1, RTN2, RTN3* and *RTN4*. Reticulons were first described in neuroendocrine cells and their function is implicated in neurodegenerative diseases. They localize primarily to the ER membrane as ER-shaping proteins. Reticulons are known to induce apoptosis, inhibit axon regeneration and regulate protein trafficking^5^,^6^,^7^,^8^,^9^. The human *RTN1* gene has three transcript variants that encode for three *RTN1* isoforms: *RTN1A*, *RTN1B* and *RTN1C*. While the function of RTN1 has been shown to be involved in endocytosis^10^, apoptosis, and regulation of ER stress and DNA damage-induced cell death^11^,^12^, the mechanism by which RTN1 exerts these effects is not known and its structure–function relationship has not yet been characterized. In addition, RTN1 has never been studied in the context of kidney disease. In the current study, we report that the expression of RTN1, specifically RTN1A isoform, is highly upregulated in both human and mouse models with kidney disease, and that its increased expression induces ER stress response and apoptosis in renal cells. Reduction of RTN1A expression *in vivo* results in attenuation of renal fibrosis and diabetic kidney injury, which is associated with decreased ER stress markers, indicating that RTN1A is a novel mediator of CKD progression that promotes renal injury through ER stress.

## Results

### Identification of *RTN1* as a highly upregulated gene in CKD

To identify genes that contribute to the development of CKD, we examined the renal gene expression of mice with varying extents of renal injury. Tg26 mice, a CKD model of HIVAN, develop significant proteinuria at 4 weeks of age. Most Tg26 mice do not survive past 3–6 months of age due to rapidly progressive renal failure. However, some Tg26 mice have milder kidney injury with stable renal function. The kidney histology of Tg26 mice is characterized by collapsing focal segmental glomerulosclerosis and significant tubular interstitial inflammation and fibrosis with tubular atrophy and dilatation^13^. To identify genes that are differentially regulated and potentially responsible for the severity of CKD in Tg26 mice, we compared the kidney transcriptomes of Tg26 mice having mild and severe kidney disease with gender-matched wild-type (WT) littermates (Supplementary Table 1). Cluster analysis of differentially expressed genes was performed among these three groups of mice by analysis of variance (ANOVA). Differentially expressed genes were then further grouped based on the patterns of change into five clusters (Supplementary Fig. 1). Cluster 5 contains a set of genes that demonstrated a pattern where the level of expression correlated with the severity of renal injury (Supplementary Fig. 1; Supplementary Table 2). Several genes in this list are known to be involved in the progression of CKD, such as *Prom1 and Tgm2* (refs 14, 15).

### *RTN1* expression correlated inversely with renal function in DN

**Figure 1:**
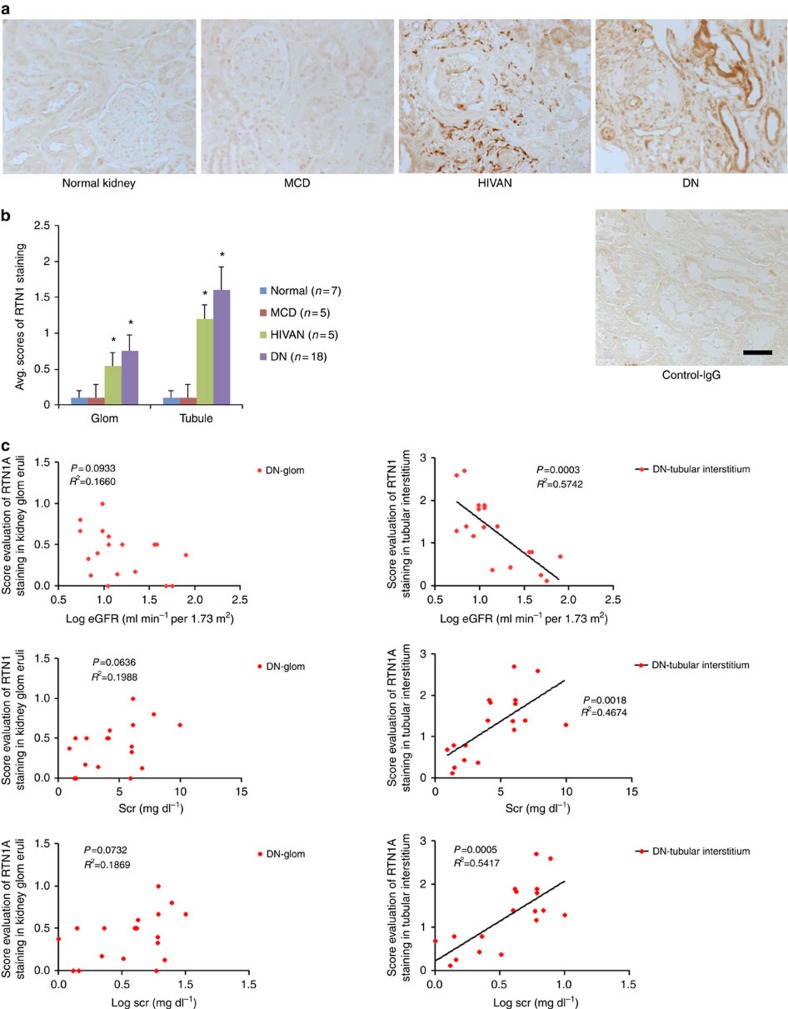
Increased RTN1A expression is associated with progression of CKD. (**a**) Representative immunostaining of RTN1A in kidney sections of patients with minimal change disease (MCD) (*n*=5), HIVAN (*n*=5) and diabetic nephropathy (DN) (*n*=18), as well as in normal kidneys of nephrectomy samples (*n*=7). Immunostaining was performed in duplicates. (**b**) Semi-quantitative scoring of RTN1A staining for both glomerular and tubular interstitial compartments summarized in a bar graph. *P<0.05, Scale bar, 50 μm. The data were expressed as mean±s.d. The ANOVA with Bonferroni correction was used. (**c**) Correlation between the intensity of RTN1A staining in glomerular or tubular compartment and renal function (eGFR or serum creatinine) was calculated in patients with DKD using Pearson and Spearman correlation analysis as described in the method. *P* and *R*^2^ are indicated on the graph. *n*=18.

To ensure candidate genes identified from the Tg26 model were relevant to human kidney disease, we examined the expression of the genes in cluster 5 in human diseased kidneys by taking advantage of the publically available data sets from Nephromine.org^16^. We found that *RTN1* expression was higher in kidneys of patients with diabetic nephropathy (DN) compared with healthy controls (Supplementary Fig. 2a). Levels of RTN1 messenger RNA (mRNA) transcripts correlated inversely and significantly with estimated glomerular filtration rate (eGFR) in this DN population (*R*=−0.56, *P*=7.8 × 10^−5^; Supplementary Fig. 2b). In addition, previous studies in the neurons suggested that RTN1 might be involved in ER stress and apoptosis, which are key pathologic processes leading to the progression of CKD^11^,^12^. Therefore, we selected RTN1 as a priority gene for our current study, as its function has never been examined in the context of CKD. To further confirm the relevance of RTN1 in kidney disease, we performed RTN1 immunostaining on kidney biopsy samples of patients with minimal change disease (MCD), DN and HIVAN as well as normal kidney sections of nephrectomy samples. Among three splice variants of RTN1, only RTN1A staining was more pronounced in DN and HIVAN kidney sections compared with MCD and normal kidney sections using an antibody raised against the residues 174–337 at the N terminal of RTN1A (Abcam ab8957), which does not recognize RTN1B or RTN1C isoforms. (Fig. 1a,b). The specificity of the staining was confirmed by negative control with IgG and baseline weak staining in the normal kidney. In addition, immunostaining with the antibodies specific for RTN1C did not reveal any changes between normal and diseased kidneys (Supplementary Fig. 3). Overall, RTN1A staining was stronger in the tubular compartment compared with the glomerular compartment. Interestingly, RTN1A staining localized more in the interstitial cells in the HIVAN kidneys compared with the DN kidneys, which may be due to the severe infiltration of RTN1A-positive inflammatory cells in the HIVAN kidneys. Similarly, in the diabetic kidneys, tissues of some patients had more staining of tubular cells, while others had more staining of interstitial cells (Supplementary Fig. 4). The differential pattern of RTN1A expression is again likely dependent on the severity of tubular cell injury and the degree of infiltration of inflammatory cells between DN patients. Semi-quantitative assessment of RTN1A staining on kidney sections from 18 patients with DN demonstrated that RTN1A staining intensity in the tubular compartment inversely correlated with eGFR and serum creatinine, while no significant correlation was observed between glomerular staining and eGFR or serum creatinine (Fig. 1c; Supplementary Table 3; Supplementary Fig. 4). Taken together, these data suggest that both mRNA and protein expression of RTN1A are associated with progression of CKD in humans.

### *Rtn1a* is upregulated in multiple murine models of CKD

**Figure 2:**
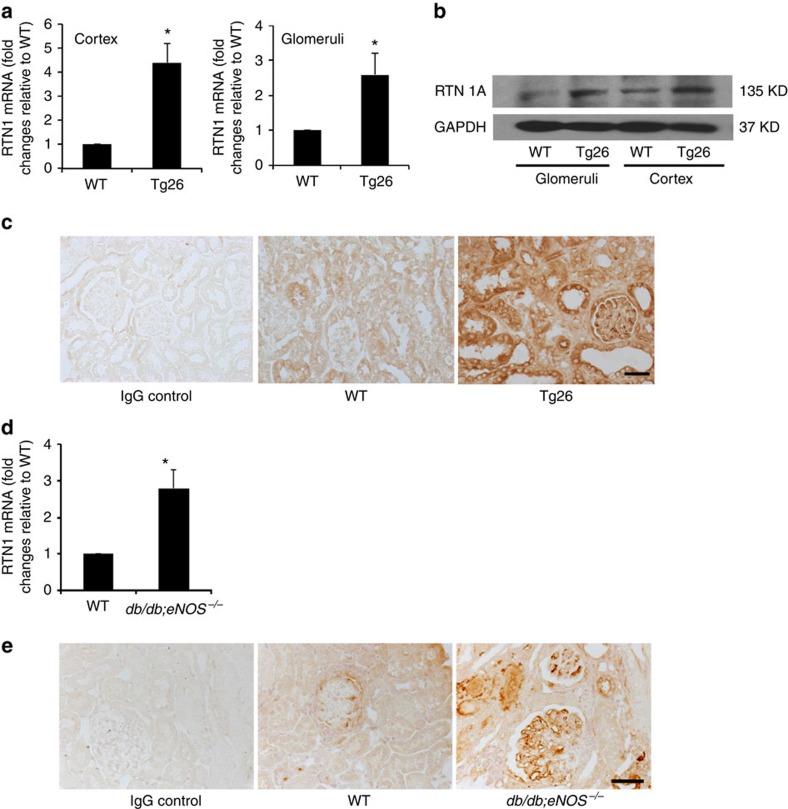
Increased RTN1A in murine CKD models. (**a**) *Rtn1a* mRNA expression in the glomeruli (Glom) and kidney cortex (Cortex) of wild-type (WT) and Tg26 mice quantified by qPCR. *n*=3, *P<0.05, compared with WT group. (**b**) Western blots of total protein lysates from Glom and Cortex. Representative blots of three independent experiments are shown. (**c**) Immunohistochemistry staining of kidney sections from Tg26 and WT control mice and representative images of three mice in each group are shown. Negative control with unrelated IgG is also shown. (**d**) mRNA expression of *Rtn1a* in the renal cortices of *db/m;eNOS*^*+/+*^ (WT) and *db/db;eNOS*^*−/−*^ mice as determined by real-time PCR. *n*=3 for each group. *P<0.05, compared with WT group. (**e**) Immunostaining of RTN1A in kidney sections of WT and *db/db;eNOS*^*−/−*^ mice and representative images of three mice in each group as well as negative controls with unrelated IgG are shown. Scale bar, 50 μm. Each PCR experiments were performed in triplicate. Western blot and immunostaining were performed in duplicate. The data were expressed as mean±s.d. The two-sided unpaired *t*-test was used.

We next examined RTN1A expression in multiple murine models of CKD. First, we confirmed that *Rtn1a* mRNA expression was indeed higher in the kidney cortex and isolated glomeruli of Tg26 mice compared with WT littermates, validating the transcriptome profile analyses (Fig. 2a). RTN1A protein level was also significantly higher in both the glomerular and tubular compartments of Tg26 mice compared with WT littermates (Fig. 2b,c). Consistent with the human data, we were not able to detect a difference in the expression of RTN1C with isoform-specific antibodies (Supplementary Fig. 5). We also did not detect RTN1B expression in the normal or diseased kidney, suggesting that RTN1B expression might be low or absent in the kidney. We therefore focused on ascertaining the role of RTN1A in kidney disease.

We next examined the renal expression of *Rtn1a* in the *db/db* model of DN with a knockout of the endothelial nitric oxide synthase gene (*Nos3*). These *db/db;eNOS*^*−/−*^ mice were killed at age of 5 months when they developed significant diabetic kidney injury. Real-time quantitative PCR (qPCR) showed that the expression of *Rtn1a* was significantly higher in kidneys of *db/db;eNOS*^*−/−*^ mice compared with nondiabetic *db/m;eNOS*^*+/+*^control mice (WT) (Fig. 2d). Interestingly, immunostaining of RTN1A was more pronounced in the glomerular area than tubular compartment of *db/db;eNOS*^*−/−*^mice compared with WT mice (Fig. 2e), which is consistent with the observation that diabetic mice have more injury in their glomeruli than tubules. Furthermore, expressions of *Rtn1a* mRNA and protein were also higher in kidneys of mice killed at 10 days after unilateral ureteral obstruction (UUO) and at 4 weeks after injection of folic acid (folic acid-induced nephropathy) compared with their respective controls (Supplementary Figs 6 and 7). Since the UUO mainly induces tubular cell injury, the staining was limited only to the tubular compartments. To further confirm the specificity of the RTN1A staining, we used a second rabbit polyclonal antibody against mouse RTN1A from a different source (CD Creative Diagnosis, Cat# DPABT-H23572). As shown in the Supplementary Fig. 8, we found a similar pattern of staining in the kidneys from mouse HIV, diabetic and UUO models using this new antibody as compared with those with the previous antibody from Abcam. These findings in multiple murine models, together with the previous data in human CKD, strongly suggest that RTN1A specifically increases in sites of renal injury in CKD, irrespective of the aetiology of the disease.

### Role of RTN1A in ER stress of kidney cells

**Figure 3:**
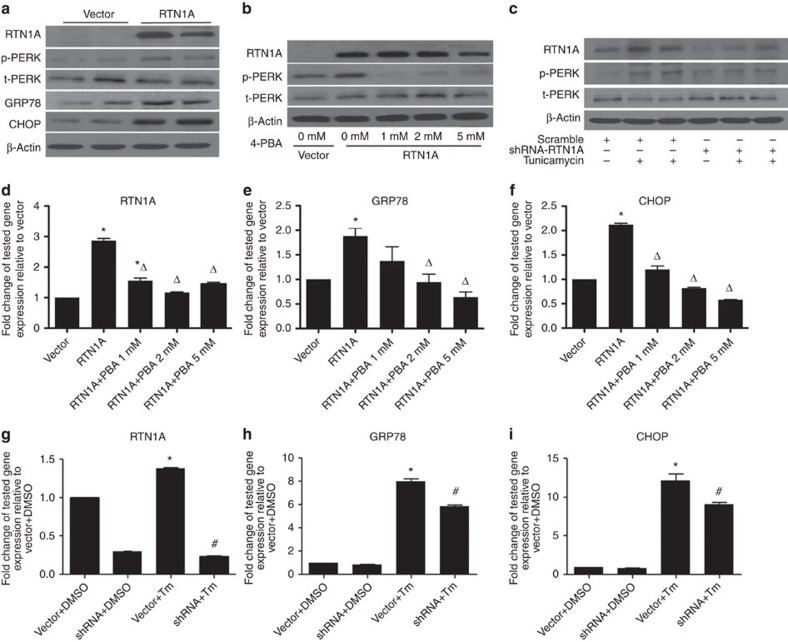
Role of RTN1A in ER stress. (**a**) HK2 cells were transfected with *RTN1A* or control vector for 3 days. Representative western blots for RTN1A, p-PERK, T-PERK (total PERK), GRP78, CHOP and β-actin are shown. (**b**) HK2 cells transfected with *RTN1A* were treated with an ER stress inhibitor 4-PBA at 0, 1, 2 and 5 mM. Representative western blots of RTN1A, p-PERK, T-PERK and β-actin are shown. (**c**) HK2 cells infected with a lentivector containing either scramble shRNA or *RTN1A*-shRNA were stimulated with tunicamycin (Tm) at 25 ng ml^−1^ or DMSO as control for 24 h. Representative western blots of RTN1A, p-PERK, T-PERK and β-actin are shown. (**d**–**f**) HK2 cells transfected with *RTN1A* or control vector were treated with an ER stress inhibitor 4-PBA at 0, 1, 2 and 5 mM. Real-time qPCR analyses for *RTN1A* and genes involved in ER stress markers (*GRP78* and *CHOP)* were performed. *P<0.05, compared with vector group, ^Δ^*P*<0.05, compared with RTN1A group, *n*=3. (**g**–**i**) HK2 cells infected with either a lentivector containing scramble (vector) or RTN1A-shRNA (shRNA) were stimulated with DMSO or tunicamycin (Tm) for 24 h. Real-time PCR analysis for *RTN1A* and genes involved in ER stress markers (*GRP78* and *CHOP)* were performed. *P<0.05 compared with vector+DMSO group, ^**#**^*P*<0.05 compared with vector+Tm group, *n*=3. Each PCR experiment was performed in triplicate and western blots were performed in duplicate. The data were expressed as mean±s.d. The ANOVA with Bonferroni correction was used.

Since RTN1A is an ER-associated protein and RTN1C has been shown previously to be involved in ER stress in neuronal cells^12^, we examined whether RTN1A also mediates ER stress response in kidney cells using overexpression and knockdown approaches. First, we confirmed the efficiency of the overexpression and short hairpin RNA (shRNA) knockdown constructs in HEK293T cells. Using an *RTN1A* expression construct, we successfully overexpressed RTN1A in HEK293T cells (Supplementary Fig. 9a,b). We also screened four different clones of shRNAs for *RTN1A* knockdown, and shRNA CL-1 and CL-4 were selected based on the degree of knockdown by western blot analysis (Supplementary Table 4; Supplementary Fig. 9c). In addition, we confirmed that these shRNAs knocked down only RTN1A isoform but not RTN1C (Supplementary Fig. 9c). The level of RTN1B was undetectable using currently available antibodies that detect all isoforms. We then used the RTN1A overexpression and knockdown approach to determine the role of RTN1A in ER stress in HK2 cells. By western blotting, we found that the overexpression of RTN1A increases PERK phosphorylation and expression of GRP78 and CHOP in HK2 cells (Fig. 3a). This increase in PERK phosphorylation by RTN1A overexpression was prevented by pretreatment with an inhibitor of ER stress, 4-phenylbutyrate (4-PBA) (Fig. 3b). Tunicamycin, which is a mixture of nucleoside antibiotics known to induce unfolded protein response and trigger ER stress in eukaryotic cells, induced RTN1A expression and PERK phosphorylation. Conversely, knockdown of *RTN1A* prevented tunicamycin-induced phosphorylation of PERK (Fig. 3c; densitometric analysis of the western blots are shown in the Supplementary Fig. 10). In addition, the overexpression of RTN1A increased the expression of ER stress markers in HK2 cells (*GRP78* and *CHOP*), which were abolished by pretreatment with 4-PBA (Fig. 3d–f). We also confirmed that tunicamycin significantly induced the mRNA expression of RTN1A as well as markers of ER stress (*GRP78* and *CHOP*) in HK2 cells, which were attenuated by knockdown of *RTN1A* (Fig. 3g–i). We then examined the time course of the effects of RTN1A overexpression on the expression of ER stress markers (Supplementary Fig. 11a) and the expression of the CHOP target genes in RTN1A-overexpressing cells (Supplementary Fig. 11b). These data further confirmed the role of RTN1A in ER stress response in the kidney cells.

### Role of RTN1A in apoptosis of HK2 cells

**Figure 4:**
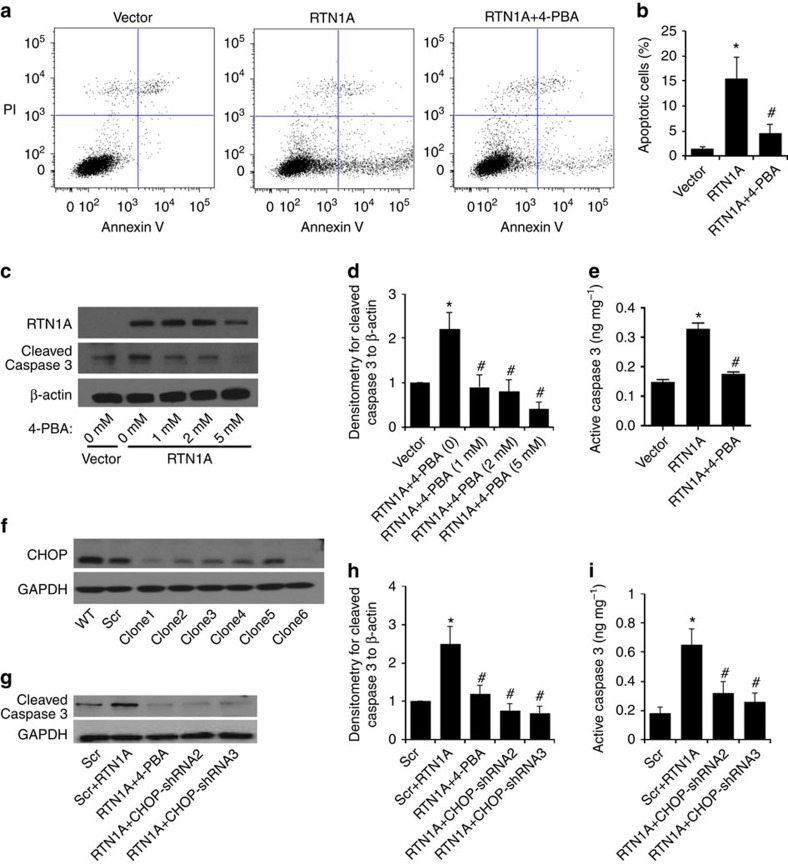
Role of RTN1A in apoptosis of HK2 cells. (**a**) Flow cytometric analysis of HK2 cells for apoptosis after double labelling with Annexin V and propidium iodide. HK2 cells were transfected with a *RTN1A* expression vector (RTN1A) or an empty vector (Vector) by electroporation 5 days before the apoptosis study. RTN1A-overexpressing cells were treated with or without 4-PBA at 5 mM 24 h post-transfection. (**b**) Summary of apoptosis data on HK2 cells. *P<0.01 compared with vector, ^**#**^*P*<0.05 compared with RTN1A cells, *n*=3. (**c**,**d**) Western blots and densitometry analysis confirmed that cleavage of Caspase-3 was increased in RTN1A overexpressed HK2 cells and inhibited by 4-PBA treatment (0, 1, 2 and 5 mM). (**e**) Caspase-3 activity was measured by enzyme-linked immunosorbent assay in HK2 cells transfected with RTN1A construct or vector for 5 days. RTN1A-overexpressing cells were treated with or without 5 mM 4-PBA for 24 h post-transfection. *P<0.05 compared with vector, ^**#**^*P*<0.05 compared with RTN1A cells, *n*=3. (**f–i**) RTN1A-induced ER stress was inhibited by knockdown of *CHOP* using specific shRNA. The efficiency of knockdown was confirmed by western blot analysis (**f**). On the basis of these data, shRNA#2 and shRNA#3 were selected. Using these shRNA, we confirmed that knockdown of *CHOP* attenuated RTN1A-induced apoptosis as assessed by western blot analysis of cleaved Caspase-3 (**g**), which is confirmed by densitometry analysis (**h**). This was also confirmed by measurement of Caspase-3 activity in these cells (**i**). *P<0.01 when compared with scramble shRNA, ^#^*P*<0.01 when compared with scramble shRNA+RTN1A overexpression, *n*=3. Each experiment of flow cytometry was performed in duplicate. Western blot was performed in triplicate. The data were expressed as mean±s.d. The two-sided unpaired *t*-test or ANOVA was used where are appropriate.

Since prolonged ER stress leads to apoptosis and previous studies suggested that RTN1 is involved in the induction of apoptosis of neuronal cells^11^,^12^, we assessed whether RTN1A mediated apoptosis of kidney cells. We found that overexpression of RTN1A-induced apoptosis of HK2 cells, as detected by Annexin V labelling (Fig. 4a,b) and Caspase-3 activation based on cleavage and activity (Fig. 4c–e). In contrast, inhibition of ER stress by 4-PBA attenuated RTN1A-induced apoptosis in HK2 cells, suggesting that RTN1A induces apoptosis through ER stress (Fig. 4a–e). Since 4-PBA may have some nonspecific effects, we further confirmed that RTN1A-induced ER stress is blocked by shRNA-mediated knockdown of *CHOP* (Fig. 4f–i). Furthermore, knockdown of *RTN1A* also attenuated tunicamycin-induced apoptosis in HK2 cells (Supplementary Fig. 12). Taken together, these data suggest that RTN1A plays a key role in ER stress-induced apoptosis of kidney cells.

### RTN1A mediates high glucose-induced ER stress in HK2 cells

**Figure 5:**
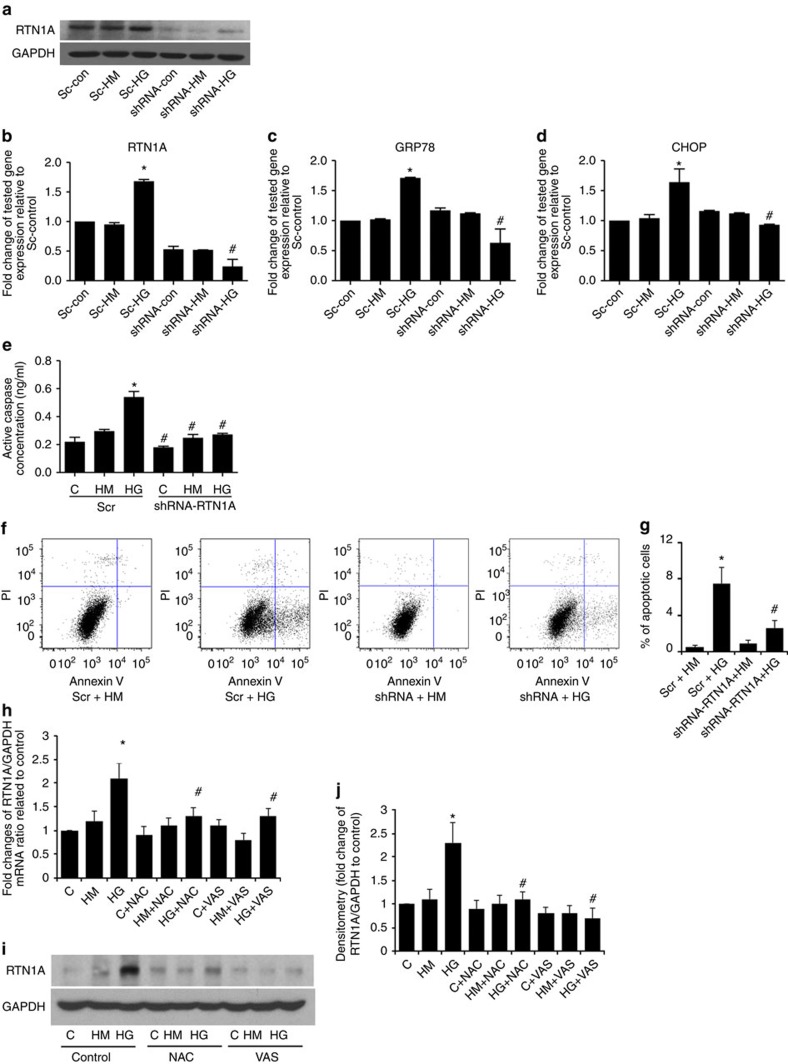
RTN1A mediates HG-induced ER stress and apoptosis in HK2 cells. (**a**) Western blots of RTN1A using HK2 cells that were transduced with lentiviral shRNA construct targeting *RTN1A* (shRNA) or scrambled shRNA (Sc) as control. Four days after infection, HK2 cells were incubated with normal glucose at 6 mM (no Tx) or normal glucose with equal osmolarity of mannitol (HM) or high glucose (HG) at 30 mM for an additional 24 h. (**b**) mRNA expression of *RTN1A* and (**c**,**d**) markers of ER stress as quantified by qPCR. After knockdown of RTN1A for 3 days, HK2 cells were incubated with normal glucose (C), normal glucose with equal osmolarity of mannitol (HM), or high glucose (HG) at 30 mM for an additional 3 days. The apoptosis was assessed by measurement of Caspase-3 activity using enzyme-linked immunosorbent assay (**e**) and flow cytometry (**f**,**g**). *P<0.05 compared with Scramble-control group, ^#^*P*<0.05 compared with Scramble-HG group, *n*=3. To assess whether reactive oxygen species mediates HG-induced RTN1A expression, we treated HK2 cells with C, HM and HG as above for 24 h with or without *N*-acetylcystein (NAC) or VAS2870 (Vas). Expression of RTN1A was assessed by qPCR (**h**) and western blot (**i**) analysis. The western blots were quantified by densitometry (**j**). *P<0.05 compared with control, ^#^*P*<0.05 compared with HG, *n*=3. Each PCR experiment was performed in triplicate. Flow cytometry and western blots were performed in duplicate. The data were expressed as mean±s.d. The ANOVA with Bonferroni correction was used for multiple group analysis.

Since high glucose is known to induce ER stress in kidney cells^17^, we examined whether RTN1A is required for high-glucose-induced ER stress in HK2 cells. We found that high glucose induced the expression of RTN1A and ER stress markers (*GRP78* and *CHOP*) in HK2 cells compared with cells treated with mannitol of the same osmolarity (Fig. 5a–d). Knockdown of *RTN1A* attenuated high-glucose-induced increase in RTN1A expression (Fig. 5a,b) and ER stress marker expression (Fig. 5c,d). Furthermore, we found that *RTN1A* knockdown inhibited the high-glucose-induced apoptosis of HK2 cells, as assessed by the measurement of Caspase-3 activity by enzyme-linked immunosorbent assay (Fig. 5e) and flow cytometry analysis using Annexin V (Fig. 5f,g). To understand how high glucose induces RTN1A expression, we treated HK2 cells with either normal glucose, normal glucose with mannitol, high glucose together with or without inhibitor of reactive oxygen species (*N*-acetylcystein), or inhibitor of NOX (VAS2870). We found that the inhibition of reactive oxygen species by either *N*-acetylcystein or VAS2870 inhibited high-glucose-induced RTN1A expression, suggesting that high glucose induces RTN1A expression likely through the activation of oxidative stress pathway (Fig. 5h,j).

### RTN1A mediates high-glucose-induced ER stress in podocytes

In addition to renal tubules, we examined the effects of RTN1 overexpression in podocytes. Human podocytes were transfected with control or RTN1A overexpression vector for 3 days and expression of ER stress markers were determined by western blot. Overexpression of RTN1A in podocytes also induced ER stress, as demonstrated by increased expression of ER stress markers (GRP78 and CHOP) (Supplementary Fig. 13a). Conversely, knockdown of RTN1A expression attenuated high-glucose-induced ER stress, as assessed by decreased *GRP78* and *CHOP* expression in podocytes (Supplementary Fig. 13b–d), suggesting that RTN1A mediates ER stress in podocytes.

### RTN1A N- and C-terminal domains are required for ER stress

**Figure 6:**
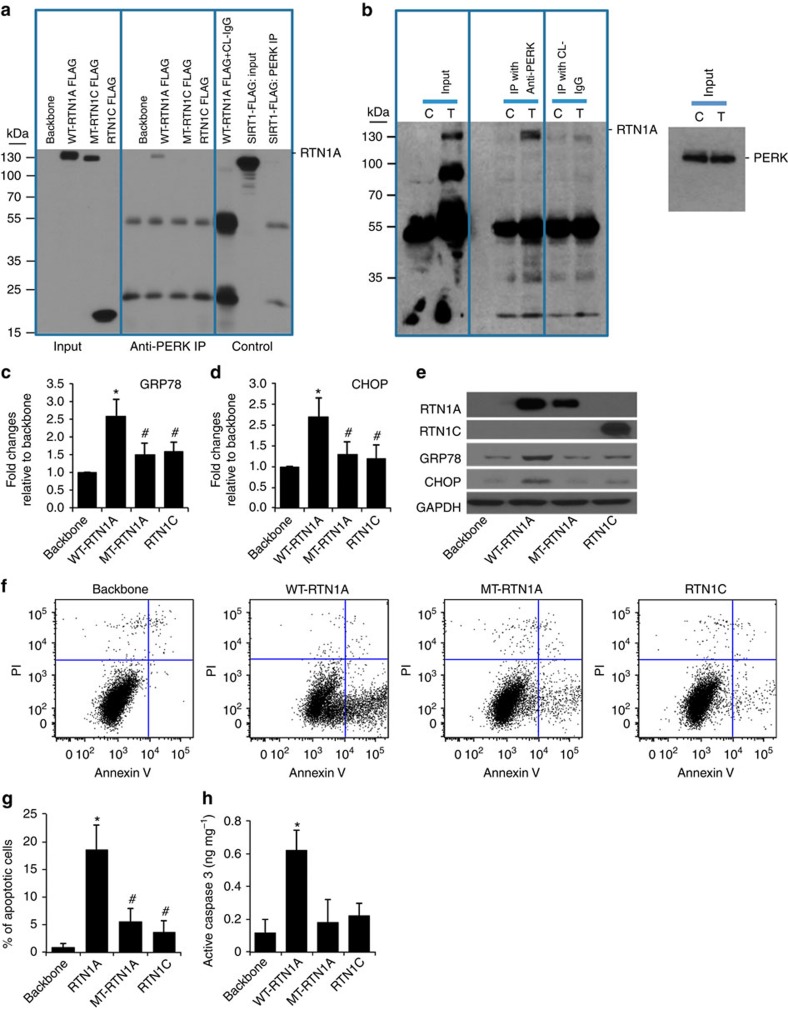
Both N and C terminals of RTN1A are required for interaction with PERK. We generated FLAG-tagged constructs of wild-type (WT) full-length *RTN1A* (RTN1A), C-terminal domain deletion mutant (MT-RTN1A) and *RTN1C* isoform, which has only C-terminal domain of *RTN1A* (RTN1C), which were used to determine whether C-terminal or N-terminal domain of RTN1A is required for its interaction with PERK. Including a nonrelevant FLAG-tagged construct (FLAG-Sirt1), all constructs were overexpressed in HK2 cells for 3 days. Cell lysates were then used for IP with anti-PERK antibody and immunoblotted with anti-FLAG antibody (**a**). For endogenous co-IP experiments (**b**), HK2 cells were treated with tunicamycin (T) or DMSO (C) for 24 h. Cells were lysed for IP with anti-PERK antibody and immunoblotted with anti-RTN1A antibody. Input lysates were also assessed by western blot analysis with anti-PERK antibody. HK2 cells were transfected with either WT-RTN1A, MT-RTN1A or RTN1C for 3 days were analysed by real-time quantitative qPCR (**c**,**d**) or western blot (**e**) for RTN1A, RTN1C, ER stress protein GRP78 and CHOP, and GAPDH. HK2 cells transfected with WT-RTN1A, MT-RTN1A or RTN1C for 5 days were used for analysis of apoptosis by flow cytometry (**f,g**) and measurement of Caspase-3 activity (**h**). *P<0.01 compared with cells transfected with the empty backbone vector, ^#^*P*<0.05 compared with WT-RTN1A, *n*=3. Each PCR experiment was performed in triplicate. Flow cytometry, IP and western blots were performed in duplicate. The data were expressed as mean±s.d. The ANOVA with Bonferroni correction was used for multiple group analysis.

RTN1 has two unusually long hydrophobic regions, separated by a 66 amino-acid long, hydrophilic loop and followed by a short C-terminal tail (as depicted in Supplementary Fig. 14). RTN1A, RTN1B and RTN1C share the same C-terminal domain, which is well-conserved in all reticulon proteins^18^, but vary in the length of their N-terminal regions, which also varies markedly between the reticulon proteins. While RTN1C has extremely short N-terminal sequences, RTN1A has a much longer N-terminal region (RTN1A is about 400 amino-acids longer than RTN1B) that is likely to confer specific biological functions. Somewhat surprisingly, no recognizable protein domains have been identified so far in the N-terminal regions^19^. Our studies suggest that among three RTN isoforms only RTN1A is induced in the diseased kidney and mediates ER stress and apoptosis in kidney cells. To explore how RTN1A induces ER stress in kidney cells, we made several constructs of RTN1A for *in vitro* functional studies. In addition to the full-length RTN1A construct (WT-RTN1A), we made a mutant construct of RTN1A with the deletion of its C-terminal domain (MT-RTN1A) and a construct for RTN1C isoform, which has a very short N-terminal domain. We then performed an immunoprecipitation (IP) assay to determine whether these RTN1A proteins interact with ER stress protein PERK. Interestingly, we found that while full-length WT-RTN1A interacts with PERK, neither MT-RTN1A nor RTN1C did not (Fig. 6a). These findings suggest that both N-terminal and C-terminal domains of RTN1A are required for its interaction with and possibly to activate PERK. In addition, we confirmed the interaction of RTN1A with PERK in kidney cells by co-IP with endogenous protein (Fig. 6b). Since endogenous RTN1A expression is low, we performed the IP of RTN1A with PERK in cells stimulated with tunicamycin to induce RTN1A expression (Fig. 6b). Furthermore, WT-RTN1A induced the expression of ER stress markers as measured by both real-time PCR (Fig. 6c,d) and western blot (Fig. 6e) analyses of GRP78 and CHOP, while MT-RTN1A or RTN1C did not. We also found that overexpression of the MT-RTN1A or RTN1C had much less effects on apoptosis of HK2 than overexpression of WT-RTN1A, as assessed by Annexin V flow cytometry (Fig. 6f,g) and measurement of Caspase-3 activity (Fig. 6h). These data suggest that both N-terminal and C-terminal domains of RTN1A are required for RTN1A-induced apoptosis of kidney cells. In the future studies, we will further determine how the interaction between RTN1A and PERK induces ER stress and apoptosis in renal cells.

### Role of RTN1 in animal models of kidney disease

To determine the functional role of RTN1 *in vivo* in kidney disease, a doxycycline (DOX)-inducible RNA interference (RNAi) model was developed^20^ for *Rtn1a* as described in Methods. We generated mice with DOX-inducible widespread *Rtn1a* knockdown and a green fluorescent protein (GFP) reporter under the control of the strong synthetic CAGs (cytomegalovirus early enhancer element and chicken beta-actin) promoter, called *CAGs;Rtn1*^*RNAi*^ mice. We compared RTN1A protein and mRNA expression in the kidney cortex of *CAGs;Rtn1*^*RNAi*^ mice with and without DOX feeding (625 mg kg^*−*1^ chow for 3 weeks starting at 6 weeks of age) and showed that DOX feeding reduced RTN1A expression but not that of RTN1C. We could not detect RTN1B in the kidney using available antibodies. We have generated two lines of *CAGs;Rtn1*^*RNAi*^ mice (Rtn1_353 and Rtn1_713 mice) (Supplementary Figs 15 and 16). DOX feeding also increased the GFP expression in both lines. The Rtn1a_353 line was used in all subsequent experiments and Rtn1_713 line was used for validation experiments. To control for nonspecific responses due to DOX feeding and shRNAmir expression, *CAGs;Luc*^*RNAi*^ mice that expresses an shRNAmir guide sequence against the firefly luciferase gene^20^ were used as controls in subsequent animal studies.

**Figure 7:**
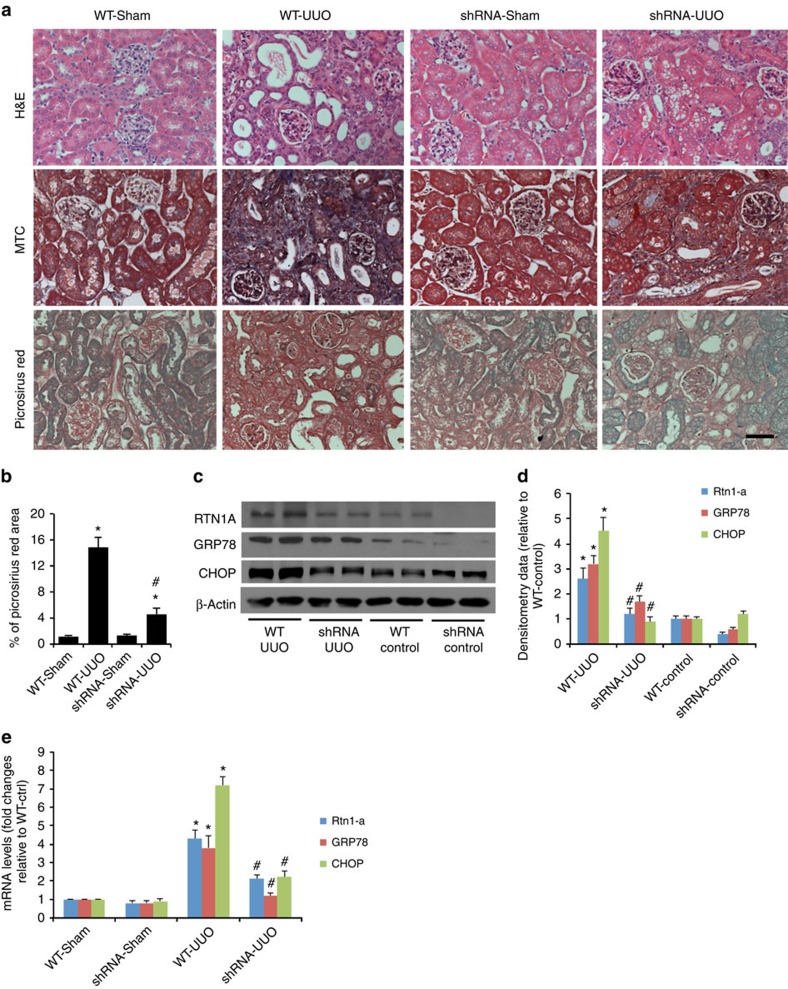
*Rtn1a* knockdown before UUO mitigates fibrosis and ER stress *in vivo.* (**a**) CAGs;Rtn1a^siRNA^ (shRNA) and CAGs;Luc^siRNA^ (WT) mice were fed with DOX for 3 weeks before the surgery. Mice were killed 10 days after the UUO or sham-operation (Ctl) and kidneys were removed for H&E, Masson's trichrome (MTC) and Picrosirius red staining. The representative pictures are shown, *n*=6. (**b**) The quantitation data of Picrosirius red staining was shown, *P<0.01 compared with respective control mice, ^#^*P*<0.05 compared with WT-UUO mice, *n*=6. (**c**–**e**) Kidney cortices of these mice were used for protein and RNA isolation. Western blots (**c**) and qPCR (**e**) were performed to analyse expression of *Rtn1a* and ER stress markers in the kidney of these mice. Western blots were analysed by densitometry (**d**). *P<0.05 compared with WT-Ctl, ^#^*P*<0.05 compared with WT-UUO, *n*=6. Each qPCR experiment was performed in triplicates. The data were expressed as mean±s.d. The ANOVA with Bonferroni correction was used.

Both *CAGs;Rtn1*^*RNAi*^ (shRNA) and *CAGs;Luc*^*RNAi*^ (WT) mice were fed with DOX from 6 to 9 weeks of age and then either the UUO or sham operation was performed at 9 weeks of age (*n*=6; 3 females and 3 males). Mice were killed 10 days after the surgery. There were no changes in body weight and urine albumin/creatinine ratio between baseline (prior DOX feeding), day of surgery or 10 days post-surgery (Supplementary Fig. 17). Both mRNA and protein levels of RTN1A increased in the UUO kidney compared to sham-operated kidney in the control mice. However, *Rtn1a* expression was suppressed in the UUO kidney of shRNA mice compared with the UUO kidney of control mice (Fig. 7c,d). Interestingly, renal fibrosis was significantly attenuated in the kidney of shRNA UUO mice as compared with those of control UUO mice (Fig. 7a,b). In addition, while PERK phosphorylation and expression of ER stress markers were increased in the kidney of control UUO mice, these were also attenuated in the kidney of shRNA UUO mice (Fig. 7c–e), suggesting that knockdown of *Rtn1a* expression improves renal fibrosis and ER stress in the UUO kidney. To confirm that the observed effects were not due to the off-target effects of shRNA, we validated these findings in another line of Rtn1a knockdown mice (Rtn1_713 line) and confirmed that knockdown of *Rtn1a* indeed attenuates renal fibrosis and ER stress marker expression in the UUO kidney (Supplementary Fig. 19).

**Figure 8:**
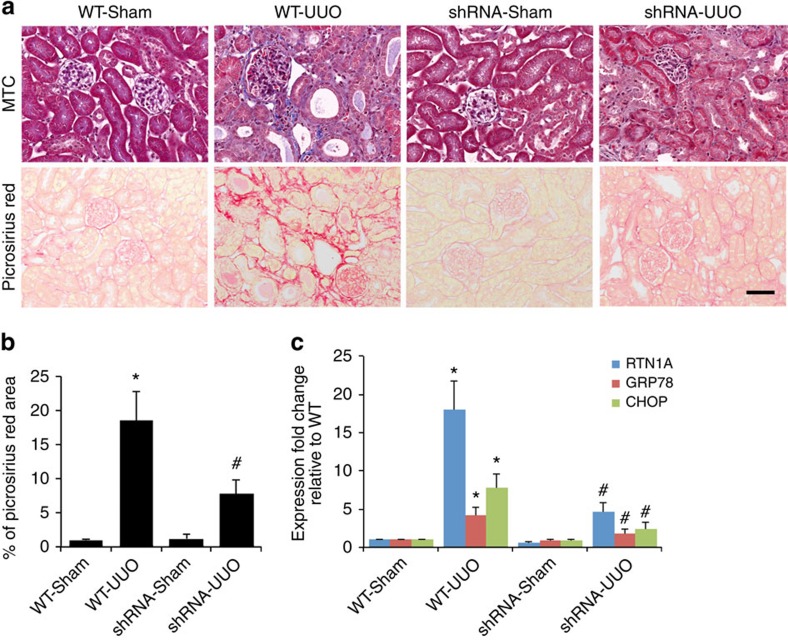
*Rtn1a* knockdown post-UUO attenuates renal fibrosis. Both *CAGs;Rtn1*^*siRNA*^ (shRNA) and *CAGs;Luc*^*siRNA*^ (WT) mice underwent either UUO or sham-operation and then fed with DOX 3 days after the surgery. The mice were killed at 21 days post-UUO. The kidneys were removed for histology analysis (**a**) and the fibrosis score was determined by quantification of Picrosirius staining (**b**). The expression of ER stress markers was analysed by qPCR (**c**). *P<0.01 compared with WT-Sham, ^#^*P*<0.05 compared with WT-UUO, *n*=6. Immunostaining was performed in duplicate. The representative pictures of six mice are shown. The data were expressed as mean±s.d. The ANOVA with Bonferroni correction was used. MTC, Masson's trichrome.

To assess whether RTN1A could be considered as a potential drug target for therapy, we next determined whether the knockdown of *Rtn1a* following kidney injury also attenuates kidney fibrosis and expression of ER stress markers. We fed the mice with DOX from post-UUO day 3 to initiate *Rtn1a* knockdown after kidney injury was established. As shown in Fig. 8, *Rtn1a* knockdown following the renal injury *in vivo* attenuated renal fibrosis and ER stress in the UUO kidneys, indicating that the inhibition of RTN1A expression is protective against kidney disease progression.

**Figure 9:**
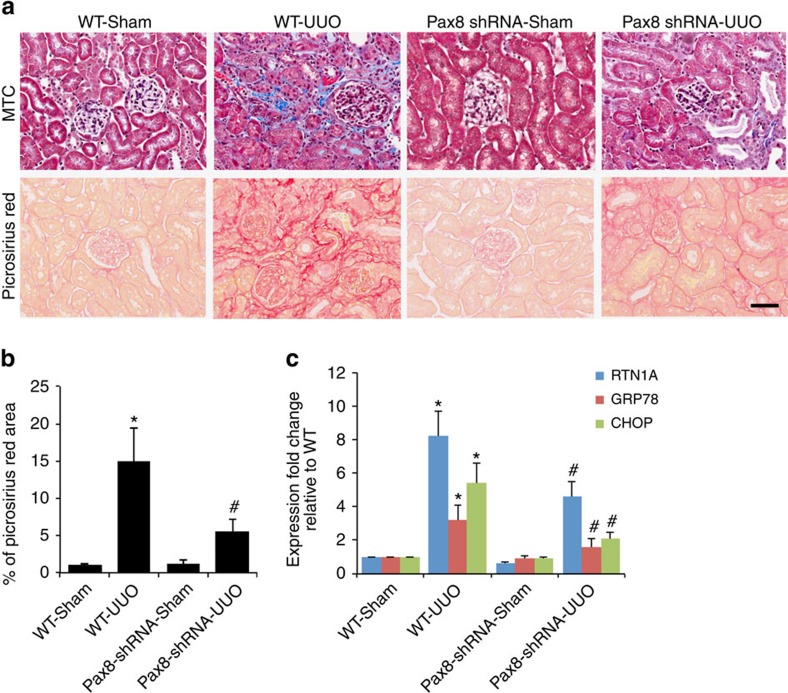
Tubule-specific *Rtn1a* knockdown attenuates renal fibrosis and ER stress. To determine the specific role of RTN1A in renal tubular cells, we generated tubular cell-specific *Rtn1a* knockdown mice (PAX8-shRNA) by crossing *Rtn1*^*siRNA*^ (shRNA) with *Pax8-rtTA* mice. PAX8-shRNA mice and their control littermates (WT) underwent UUO or sham-operation and killed 10 day after the surgery. The kidneys were removed for histology analysis (**a**). The renal fibrosis score was determined by quantification of Picrosirius red staining (**b**). The expression of ER stress markers was determined by real-time PCR analysis in these kidneys. *P<0.01 compared with WT-sham, ^#^*P*<0.05 compared with WT-UUO, *n*=6. Immunostaining was performed in duplicate. The representative pictures of six mice are shown. The data were expressed as mean±s.d. The ANOVA with Bonferroni correction was used. MTC, Masson's trichrome.

To confirm a local and direct role of RTN1A in renal tubular cells, we crossed *Pax8*-reverse tetracycline transactivator (*Pax8*-rtTA) mice with Rtn1^RNAi^ mice to generate a tubular epithelial cell-specific *Rtn1a* knockdown mouse model. In this model, we also confirmed that knockdown of *Rtn1a* in tubular cells attenuated renal fibrosis and ER stress in the UUO kidney (Fig. 9).

**Figure 10:**
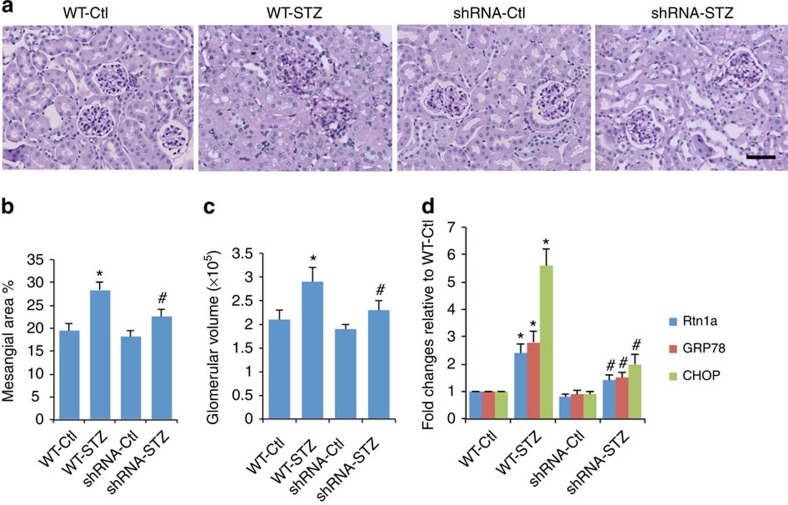
*Rtn1a* knockdown protects against DN development. CAGs;Rtn1a^siRNA^ (shRNA) and CAGs;Luc^siRNA^ (WT) mice were injected with STZ to induce diabetes at age of 8 weeks. Mice were fed with DOX from 10 to 24 weeks and killed at age of 24 weeks. Urine albumin was determined (Supplementary Table 5). Kidneys were removed for histology (**a**) and glomerular volume (**b**) and mesangial area (**c**) were quantified. (**d**) Renal expression of *Rtn1a* and ER stress markers was determined by real-time PCR. The representative pictures of kidney histology are shown. *P<0.05 compared with nondiabetic WT mice (WT-Ctl), ^#^*P*<0.01 compared with diabetic WT mice (WT-STZ), *n*=6. Each PCR experiment was performed in triplicates. The data were expressed as mean±s.d. The ANOVA with Bonferroni correction was used.

Finally, we examined whether knockdown of *Rtn1a* affects the progression of DN. Both *CAGs;Rtn1*^*RNAi*^ (shRNA) and *CAGs;Luc*^*RNAi*^ (WT) mice were injected intraperitoneally with low doses of streptozotocin (STZ) to induce diabetes at 8 weeks of age (*n*=6; 3 females and 3 males). Blood glucose levels were monitored weekly. Mice were fed with DOX from 10 to 24 weeks of age when they were killed. Compared with nondiabetic WT mice, diabetic WT mice developed significant albuminuria, kidney and glomerular hypertrophy, and mesangial expansion, which are typically seen in early DN. However, diabetic shRNA mice had attenuated albuminuria, kidney and glomerular hypertrophy, and mesangial expansion compared with diabetic WT mice (Supplementary Table 5; Fig. 10a–c), suggesting that the knockdown of *Rtn1a* also prevented the development of early DN in diabetic mice. In addition, we found that expression of *Rtn1a* and ER stress markers was induced in diabetic WT mice but inhibited in diabetic shRNA mice (Fig. 10d). These data suggest that knockdown of *Rtn1a* expression prevents early DN likely through inhibition of diabetic-induced ER stress in kidney cells. Future studies are required to determine whether knockdown of *Rtn1a* expression halts the progression of kidney disease in an animal model with progressive DN.

## Discussion

Despite optimal medical therapy, many patients with CKD progress to end-stage renal disease^4^. Therefore, it is critical to identify the underlying mechanisms mediating progression of kidney disease. In the current study, we used the Tg26 model as an experimental model of CKD to identify candidate genes that may be important for the development and progression of nephropathy. Since Tg26 mice exhibit variable severity of renal phenotypes ranging from rapid progression to renal failure (over 2–3 months) to mild disease with stable renal function for >6 months, it is an ideal system for studying factors that dictate kidney disease progression^13^. We identified a cluster of genes that were upregulated in kidneys with more severe kidney disease. This list included several interesting genes, some of which are known to be involved in the pathogenesis of CKD. We selected *RTN1* as a priority gene for our study for following reasons: (1) by searching the publically available data sets from Nephromine.org, we found that RTN1 was highly expressed in human diabetic kidney and correlated with the progression of human DN; (2) previous studies in the neurons suggested that RTN1 might be involved in ER stress and apoptosis, which are key pathologic processes leading to the progression of DN; and (3) RTN1 has never been studied in the context of kidney disease. In this study, we were able to validate the increased expression of RTN1A in human DN and HIVAN as well as in several animal models of kidney disease, indicating that RTN1A function is involved in general progression of CKD.

On injury, upregulation of RTN1A is found predominantly in the tubulointerstitial compartment including both tubular and interstitial cells. However, glomerular expression of RTN1A was also present and was associated with increased glomerular injury. These data suggest that RTN1A plays a role in both glomerular and tubular cell injury. Increased RTN1A expression is also found in interstitial cells, suggestive of its possible role in renal inflammation, which would be an important avenue to explore in future studies. Consistent with the transcriptomic data from patients with DN^16^, we confirmed that increased RTN1A protein expression in the tubulointerstitial compartment correlated with decreased kidney function in an independent cohort of patients with DN. These data strongly support a role for RTN1A in the progression of human DN.

RTNs have previously been examined in the context of neurodegenerative diseases^5^,^6^,^7^,^8^,^9^,^21^,^22^ and are known to be involved in endocytosis^10^ and initiation of apoptosis through regulation of ER stress in neuronal cells^11^,^12^. However, a biologic function of RTN1A has not been well characterized. Here we demonstrated that RTN1A plays a key role in ER stress and apoptosis of kidney cells. Our results suggest that RTN1A mediates high-glucose-induced ER stress and apoptosis of kidney cells. In addition, we found that high glucose induces RTN1A expression in kidney cells through the activation of oxidative stress pathway. These data suggest that RTN1A could be important for hyperglycaemia-induced tubular injury and progression of renal injury in kidney diseases.

To understand how RTN1A contributes to ER stress, we examined the interaction of RTN1A with PERK, a key ER stress molecule leading to the activation of apoptosis pathway. Interestingly, we found that only WT-RTN1A interacted with PERK, whereas MT-RTN1A or RTN1C did not. In addition, expression of MT-RTN1A or RTN1C was unable to induce ER stress and apoptosis in kidney cells, suggesting that both N and C terminals of RTN1A are required for its interaction with PERK and for its induction of ER stress and apoptosis in kidney cells. However, our findings are not consistent with the previous reports showing that RTN1C overexpression induces ER stress and apoptosis in neuronal cells^11^,^12^. While it is plausible that different isoforms may have cell type-specific functions, detailed mechanism of this discrepancy/inconsistency requires further studies. Future studies are also required to map the potential interactive domains between RTN1A and PERK and to understand how this interaction leads to the activation of ER stress in kidney cells.

To confirm the role of RTN1A in the progression of kidney disease, we developed a mouse model with knockdown of *Rtn1a* expression by shRNA. We found that knockdown of *Rtn1a* expression before the surgery attenuated renal fibrosis and ER stress in the UUO model. We also found that the knockdown of RTN1A expression in the mice with established kidney disease also ameliorated the progression of the disease. To verify that our findings are not a result of nonspecific off-target effects of shRNA, we validated these findings in another line of *Rtn1a* knockdown mice. In addition, we showed that tubular cell-specific knockdown of RTN1A also attenuated kidney injury, indicating an important/a direct role of RTN1A in tubular cell injury. Finally, knockdown of *Rtn1a* also ameliorated albuminuria, kidney and glomerular hypertrophy, and mesangial expansion in STZ-induced diabetic mice, suggesting that RTN1A is involved in mediating these changes of early DN. Future studies are required to confirm whether lack of RTN1A also attenuates kidney injury in an animal model of progressive DN. Taken together, this is the first *in vivo* evidence suggesting that RTN1A contributes to the development of kidney disease and that it may be a potential drug target for therapy for CKD progression.

A large body of evidence suggests that ER stress plays a major role in the development and progression of kidney disease including DN^23^,^24^,^25^. High glucose concentrations induce ER stress and apoptosis of kidney cells^17^. Diabetic rats exhibit enhanced kidney cell apoptosis, CHOP, JNK and Caspase-12 expression^26^. Diabetic *CHOP* knockout mice seemed to be protected from DN, because they developed less proteinuria than WT control mice^27^. Elevated urinary protein excretion in humans is known to be associated with tubular injury and ER stress^17^,^28^,^29^. In humans and rats with nephrotic syndrome, ER stress markers are elevated in tubular epithelial cells^30^,^31^. In addition, renal expression of ER stress markers in patients with established DN is higher than patients with mild DN. These results suggest an activation of ER stress response in the kidney of patients with established DN^17^. Over the past decade, there has been considerable interest in developing compounds that modulate ER stress response. Chemical chaperones that improve ER folding capacity, such as 4-PBA, taurine-conjugated ursodeoxycholic acid and the ER chaperone ORP150, have been shown to reduce ER stress, restore glucose tolerance and improve insulin action and sensitivity^32^,^33^,^34^. 4-PBA is an aromatic short chain fatty acid that has chaperone-like activities and recent work shows that PBA attenuates kidney injury and oxidative stress in rats with DN^35^. Our data suggest that 4-PBA abolishes RTN1A-induced ER stress and apoptosis in HK2 cells. However, the ER stress response could have both protective and deleterious features depending on whether it is the initial adaptive response or whether prolonged and chronic. An improved understanding of the molecules regulating these processes in a cell- and disease-specific manner will help identify novel therapeutic strategies targeting ER stress to prevent the progression of kidney^36^disease.

In conclusion, we have shown that RTN1A expression is highly upregulated in HIVAN and DN, and that RTN1A is a key molecule mediating hyperglycaemia-induced ER stress and apoptosis of renal cells, contributing to the progression of kidney disease. Importantly, the inhibition of its expression attenuates renal fibrosis and diabetic kidney injury, suggesting that it may be a novel therapeutic target for treatment of CKD including DN.

## Methods

### Animal studies

All animal studies were approved by the IACUC committee of Mount Sinai School of Medicine. Genomic DNA extracted from tail clipping was used. To detect the HIV-1 transgene (*Tg26*), we performed standard PCR procedures using the following primers: forward 5′- ACATGAGCAGTCAGTTCTGCCGCAGAC -3′, reverse 5′- CAAGGACTCTGATGCGCAGGTGTG -3′. Thermo profile for the PCR reaction was as follows: 95 °C for 5 min followed by 35 cycles at 95 °C for 30 s, 62 °C for 45 s and 72 °C for 45 s, then 72 °C for 10 min. Only male heterozygous Tg26 in the FVB/N background were used in the study, since homozygous Tg26 mice are not viable for more than few weeks postnatally. Male diabetic *db/db*, nondiabetic *db/m* and *eNOS*^*−/−*^ mice in B6 background were obtained from Jackson Laboratory and crossed to generate *db/db;eNOS*^*−/−*^mice and litter-matched control mice. The kidneys were collected from these mice for histology, western blot, real-time PCR analysis and microarray studies. Kidney disease was confirmed by measurement of proteinuria, renal function and histologic analysis.

### Creation of *Rtn1* knockdown mouse model

We developed a DOX-inducible RNAi model^20^ for *Rtn1*. Nine shRNA guide sequences predicted to target *Rtn1* for knockdown were embedded into a miR30-based expression cassette of a retroviral DOX-inducible shRNA vector. Two sequences achieved >50% knockdown compared with a control shRNA based on both real-time PCR and western blot analyses (Supplementary Figs 13 and 14). Two lines of genetically engineered mice, each corresponding to one of the two guide sequences that achieved >50% knockdown *in vitro*, were generated using the *ColA1 Flp/FRT* recombinase-mediated targeting system. Details of construct design and transgenesis have been described previously^20^. Briefly, the ‘Flp-In' targeting vector, called pCol-TGM, was configured with a GFP ‘spacer' between a tetracycline-regulated element and the miR30-based expression cassette. Genetically engineered ‘KH2' embryonic stem cells were used for targeted insertion of pCol-TGM to generated *TGM* mice. To generate *CAGs;Rtn1*^*RNAi*^ mice, *TGM* mice were bred with another line of genetically engineered mice that express the rtTA under the control of the CAGs promoter. *CAGs;Rtn1*^*RNAi*^ mice derived from KH2 ES cells are on a hybrid genetic background with contributions from C57BL/6 and 129/SV strains. To generate renal tubular epithelial cell-specific *Rtn1a* knockdown mice, we crossed *Pax8*-rtTA mice with *Rtn1*^*RNAi*^mice to have *Pax8;Rtn1*^*RNAi*^mice. To induce Rtn1a knockdown mice were fed 625mg/kg DOX-supplemented chow (Bio-Serv, Frenchtown, NJ) for the duration specified in the Results and figure legends.

### Creation of UUO model

To create the UUO model, the left ureter of mice was exposed through a mid-abdominal incision and ligated using 4-0 silk. Sham-operated mice had their ureters exposed and manipulated but not ligated. All surgeries were performed under general anaesthesia with isofluorane. Six mice in each group were selected based on previous studies without the use of randomization and blinding^37^.

### Creation of diabetic model by STZ injection

Induction of diabetes using STZ has been described^38^. Briefly, 8-week-old WT and shRNA mice were given low-dose STZ (50 mg kg^−1^) in 50 mmol l^−1^ sodium citrate buffer (pH 5.4) intraperitoneally daily for 5 days. At 2 weeks post-injection, mice were deprived of food for 6 h and fasting blood glucose level from the tail vein was measured using the One Touch Blood Glucose Monitoring System. Repeat fasting blood glucose measurements were taken every 2 weeks to verify hyperglycaemia. Diabetes mellitus was defined as sustained fasting blood glucose above 250 mg dl^−1^ at two distinct time points. All mice were killed at 6 months of age. Six mice in each group were selected based on our previous publication without the use of randomization and blinding^38^.

### Urine albumin and creatinine

Twenty-four-hour urine was collected from mice using metabolic cages and urine albumin was determined using a commercial assay from Bethyl Laboratory Inc. (Houston, Texas, USA). Urine creatinine levels were measured in the same samples using the QuantiChrom Creatinine Assay Kit (DICT-500) (BioAssay Systems) according to the manufacturer's instructions. The urine albumin excretion rate was expressed as the ratio of albumin to creatinine.

### Kidney histology

Kidney samples were fixed in 10% formalin, embedded in paraffin and sectioned to 4-μm thickness. Periodic acid–Schiff (PAS)-stained sections were used to assess kidney histological features. The glomerular volume and mesangial area were determined by examining PAS-stained sections using a digitizing tablet and video camera. Briefly, digitized images were scanned and profile areas were traced using ImageJ. The mean glomerular tuft volume was determined from the mean glomerular cross-sectional area by light microscopy. The glomerular cross-sectional area was calculated on the basis of the average area of 30 glomeruli in each group, and glomerular tuft volume was calculated using the following equation: GV=(*β*/*κ*) × GA^3/2^, where *β*=1.38, the shape coefficient of spheres (the idealized shape of glomeruli) and *κ*=1.1, the size distribution coefficient. Also, mesangial expansion was defined as a PAS-positive and nuclei-free area in the mesangium. Quantification of mesangial expansion was based on a minimum^39^of 10 glomeruli per section in a blinded fashion, under 400 × magnification (Zeiss AX10 microscope, Carl Zeiss Canada Ltd, Toronto, ON, Canada). The relative mesangial area was expressed as mesangial/glomerular surface area (%).

### Cell culture

HK2 and HEK293T cells were obtained from ATCC (CRL-2,190 and CRL-3,216, respectively) and cultured according to their^40^specifications. Cells were stimulated with TM (Sigma-Aldrich) with or without 4-PBA (Sigma-Aldrich) as described in the legends. Conditionally immortalized human podocytes^41^ were obtained from Dr Moin Saleem, Bristol, UK. Cells were grown in RPMI-1,640 medium supplemented with 10% fetal bovine serum and 1% penicillin–streptomycin antibiotics. Cells were cultured on rat tail collagen-I-coated plates and maintained at 33 °C (5% CO_2_, 90% humidity). Before experiments, cells were moved to a 37 °C incubator and cultured for at least 7 days to fully induce differentiation. Endotoxin-free human serum albumin was used (Sigma). Endotoxin levels in preparations were further determined by Associates of Cape Cod (Falmouth, MA, USA) and were found to be <2 endotoxin units per mg of protein.

### Transfection

The Lonza's Nucleofector Technology (Amaxa Basic Neucleofector kit for Primary Mammalian Epithelial Cells, Program T20) was used to transfect overexpression and shRNA knockdown constructs into HK2 cells with an efficiency of 80–90% based on GFP expression^42^. HEK293T cells were transfected using PolyJet transfection reagent (SignaGen Laboratories). Overexpression vector of *RTN1A* was obtained from Thermo Scientific and different clones of shRNA for *RTN1* were obtained from Open Biosystems. The efficiency of overexpression and knockdown were confirmed by both western blot and real-time PCR. The expression constructs for WT-RTN1A-FLAG, MT-RTN1A-FLAG with deletion of C-terminal domain (amino acid 1–727), and RTN1C-FLAG were generated using a PCR-based mutagenesis method. To generate the human RTN1A-FLAG WT construct, we used the following primers:

5′- TGCTAGCCACCATGGCCGCGCCGGGGGATCCACAGCGAGCTCATCATCG -3′ and 5′- AGGTCGACATCGA*TTATTTGTCATCGTCGTCCTTGTAGTCCAT***GATATC**CTCAGCGTGCCTCTTAGC -3′. The FLAG sequence is shown in italic, introduced restriction enzyme sites is in bold and stop code is in underline. The PCR products were purified and then ligated into pGEM-T easy Vector (Promega). After amplification, the DNA fragment was excised from the pGEM-T easy with NheI and NotI and then ligated into pEGFP-N1 (Clontech) at the same restriction enzyme sites. For MT-RTN1A, the primers used are: 5′- TTA**CTCGAG**TTCCAACCAAAGTCCTG -3′ and 5′- TTT**GATATC**AGCCATGAGCAGCAGGGTCAG -3′. For cloning human RTN1C-FLAG, the primers used are 5′- TA**GCTAGC**CACCATGCAGGCCACTGCCGATTCCACCAAGATGGACTGTGTGTGGAGCAACTGGAAAAGTCAGGCTATTGACCTGTTGTATTGG -3′.

### Lentiviral preparation and infection

HEK293T cells were transfected with either lentiviral plasmid expressing RTN1 shRNA sequence pGIPZ-shRTN1 (Open biosystems) or control scrambled sequence, pGIPZ-scramble, plus psPAX2 packaging plasmid and pMD2.G envelop plasmid using PolyJet transfection reagent according to the manufacturer's protocol (SignaGen Laboratories). Forty-eight hours after transfection the lentiviral particles were harvested from HEK293T cell culture medium. Concentrated lentiviral particles were used to infect HK2 cells or podocytes.

### Apoptosis analysis

Apoptosis was measured by flow cytometry using Annexin V-FITC apoptosis detection kit (BD Bioscience). Annexin V-FITC staining detects early stage apoptosis. Necrotic or late-stage apoptotic cells were labelled by propidium iodide. The number of cells labelled with Annexin V-FITC and propidium iodide was quantified using the FACS Caliber Flow cytometer and the data were analysed with CellQuest software (BD Biosciences). Caspase-3 activity was measured in HK2 cells using a Human Active Caspase-3 Immunoassay Kit (R&D Systems, Inc.) following the manufacturer's protocol.

### Real-time PCR

Samples were stored in RNAlater (Qiagen) solution at −80 °C until processing. Total RNA was extracted from isolated glomeruli or cultured podocytes using RNeasy Mini Kit (Qiagen). Superscript III First-Strand Synthesis SuperMix (Invitrogen) was used for reverse transcription of 1 μg of total RNA. PCR was performed using SYBR Green Master Mix (Applied Biosystems) and the Applied Biosystems 7500 Real-time PCR system. Intron-spanning primer sets selectively targeting mRNA and not genomic DNA sequence were designed using Primer-BLAST (NCBI) (Supplementary Table 6). The homogeneity and the size of PCR amplicons were confirmed by both melting curve analysis and agarose gel electrophoresis. Data were analysed by the 2^*−*ΔΔCT^ method^43^ and presented as fold change relative to a control sample after normalization against the expression of a housekeeping gene.

### Western blot

Tissue or cells were lysed with a buffer containing 1% NP40, a protease and phosphatase inhibitor cocktail. After protein concentration determination, cell lysates were^44^subjected to western blot analysis using specific antibodies (Supplementary Table 7).

### Immunoprecipitation

HK2 cells were transfected with FLAG-tagged WT-RTN1A, MT-RTN1A or RTN1C for 3 days. Cells were lysed with above lysis buffer and incubated with anti-PERK antibody (Cell Signaling) for overnight at 4 ^o^C and the precipitated materials were used for western blot analysis using anti-FLAG antibody (Sigma). For co-IP with endogenous protein, HK2 cells were treated with tunicamycin at 25 ng ml^−1^ or dimethylsulphoxide as control for 24 h. Then, cells were lysed as above and incubated with anti-PERK antibody for IP and then western blot analysis with anti-RTN1A antibody as above.

### Microarray studies

Affymetrix gene expression microarrays were performed at the Mount Sinai Institution Microarray Core Facility. The Affymetrix GeneChip Mouse Genome 430 2.0 Array was used to profile gene expression in the kidney cortex of Tg26 and WT mice^37^. One-way ANOVA was applied to the data set to identify the genes that were differentially expressed between the two groups. *P* values were corrected using Benjamini–Hochberg false discovery rate with a threshold of 0.05. Microarray data have been deposited in NCBI GEO under accession code GSE69074.

### Immunostaining of kidney sections

Archival human kidney biopsies were collected at Jacobi Medical Center and Mount Sinai Hospital under protocols approved by their Institutional Review Boards. Biopsy samples included 5 cases of HIVAN, 5 MCD, 18 DN and 7 normal tissues from nephrectomy samples. Five samples were used to detect the average expression level of RTN1A in the specific kidney disease. More DN patients were selected from both Jacobi Medical Center and Mount Sinai Hospital for establishing a correlation between RTN1A expression and the severity of kidney disease in this patient population. We obtained only seven normal tissues from nephrectomy samples. Immunostaining was performed using specific primary antibodies (Supplementary Table 7) and biotinylated secondary antibodies (Vector Laboratories Inc.). Staining was revealed with avidin–peroxidase (VECTASTAIN Elite; Vector Laboratories Inc.). Slides were mounted in Aqua Poly/Mount (Polysciences Inc.) and photographed under an Olympus BX60 microscope with a digital camera. The following antibodies were used: RTN1A (Abcam). The extent of kidney staining in human biopsies was semi-quantitatively scored. The staining of each glomerulus was scored in a scale of 0–4 by two independent investigators (score 0: absence of specific staining; score 1: <25% area has specific staining for RTN1A; score 2: 25–50%; score 3: 50–75%; and score 4: >75%). The average score of an individual patient was calculated by adding of all scores from individual glomeruli divided by the number of glomeruli in the kidney section. The average score of RTN1A staining and eGFR or Scr from individual patients were used for calculation of correlation in patients with DN using the Pearson and Spearman method.

### Statistical analysis

Data expressed as mean±s.d. (*X*±s.d.). The two-sided unpaired *t*-test was used to analyse data between the two groups after determination of data distributions and variance. The ANOVA with Bonferroni correction was used when more than two groups were present. Statistical significance was considered when *P*<0.05.

## Additional information

**Accession codes**: Microarray data have been deposited in NCBI GEO under accession code GSE69074.

**How to cite this article:** Fan, Y. *et al*. RTN1 mediates progression of kidney disease by inducing ER stress. *Nat. Commun.* 6:7841 doi: 10.1038/ncomms8841 (2015).

## Supplementary Material

Supplementary Figures and Tables Supplementary Figures 1-20
