# Erratum: RTN1 mediates progression of kidney disease by inducing ER stress

**DOI:** 10.1038/ncomms9710

**Published:** 2015-10-22

**Authors:** Ying Fan, Wenzhen Xiao, Zhengzhe Li, Xuezhu Li, Peter Y. Chuang, Belinda Jim, Weijia Zhang, Chengguo Wei, Niansong Wang, Weiping Jia, Huabao Xiong, Kyung Lee, John C. He

*Nature Communications* 6: Article number: 784110.1038/ncomms8841 (2015); Published: 07312015; Updated: 10222015.

The original version of the Supplementary Information attached to this Article did not contain Supplementary Tables 1–7. The HTML has now been updated to include a corrected version of the Supplementary Information.

