# Supplementary material for: RTN1 mediates progression of kidney disease by inducing ER stress
Source: Nat Commun. 2015 Jul 31;6:7841. doi: 10.1038/ncomms8841 (PMC4532799; doi:10.1038/ncomms8841)
Supplement: Supplementary Figures and Tables — Supplementary Figures 1-20 [file ncomms8841-s1.pdf]

SUPPLEMENTARY INFORMATION

Supplementary Figure 1

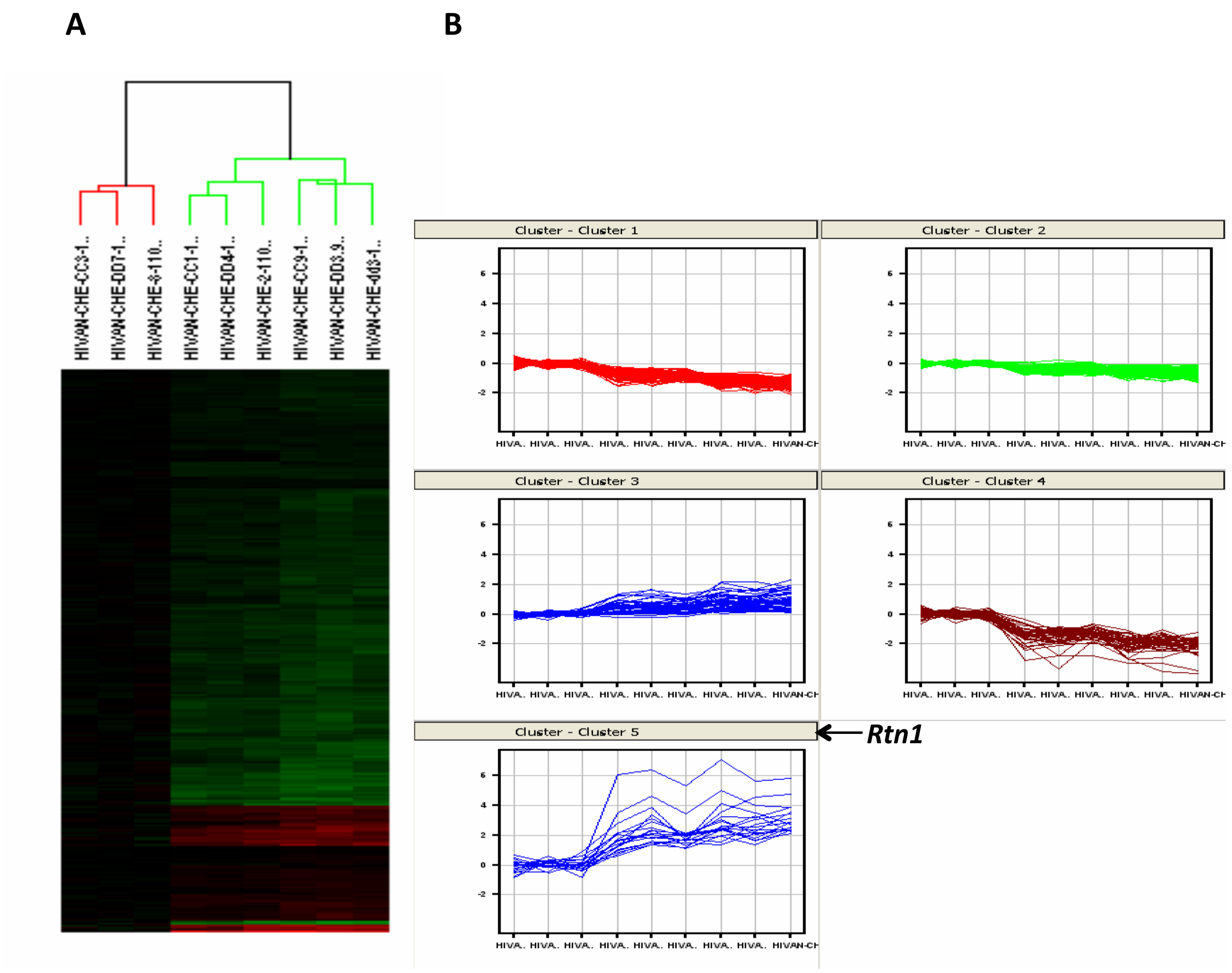

Supplementary Figure 1: Cluster analysis of microarray data from kidneys of Tg26 mice with mild and severe kidney injury as compared to the littermates.

Microarray was performed in kidneys of Tg26 with mild, severe kidney injury, and normal littermates (n=3 each group). **A.** Differentially expressed genes were identified and Heat Map of these genes is shown. **B.** Differentially expressed genes were further analyzed by ANOVA and grouped based on the patterns of changes. 5 clusters of genes with distinct patterns of changes were identified. Each intercept on the X-axis represents one mouse. There were 3 normal mice, 3 mice with mild kidney injury, and 3 mice with severe kidney injury. Y-axis indicates the fold changes of gene expression related to the average levels in 3 normal mice. Rtn1 is among the genes in the Cluster 5, whose expression progressively increases from normal to mild and to severe disease. The list of genes in the Cluster 5 is shown in the Supplementary Table 2.

Supplementary Figure 2

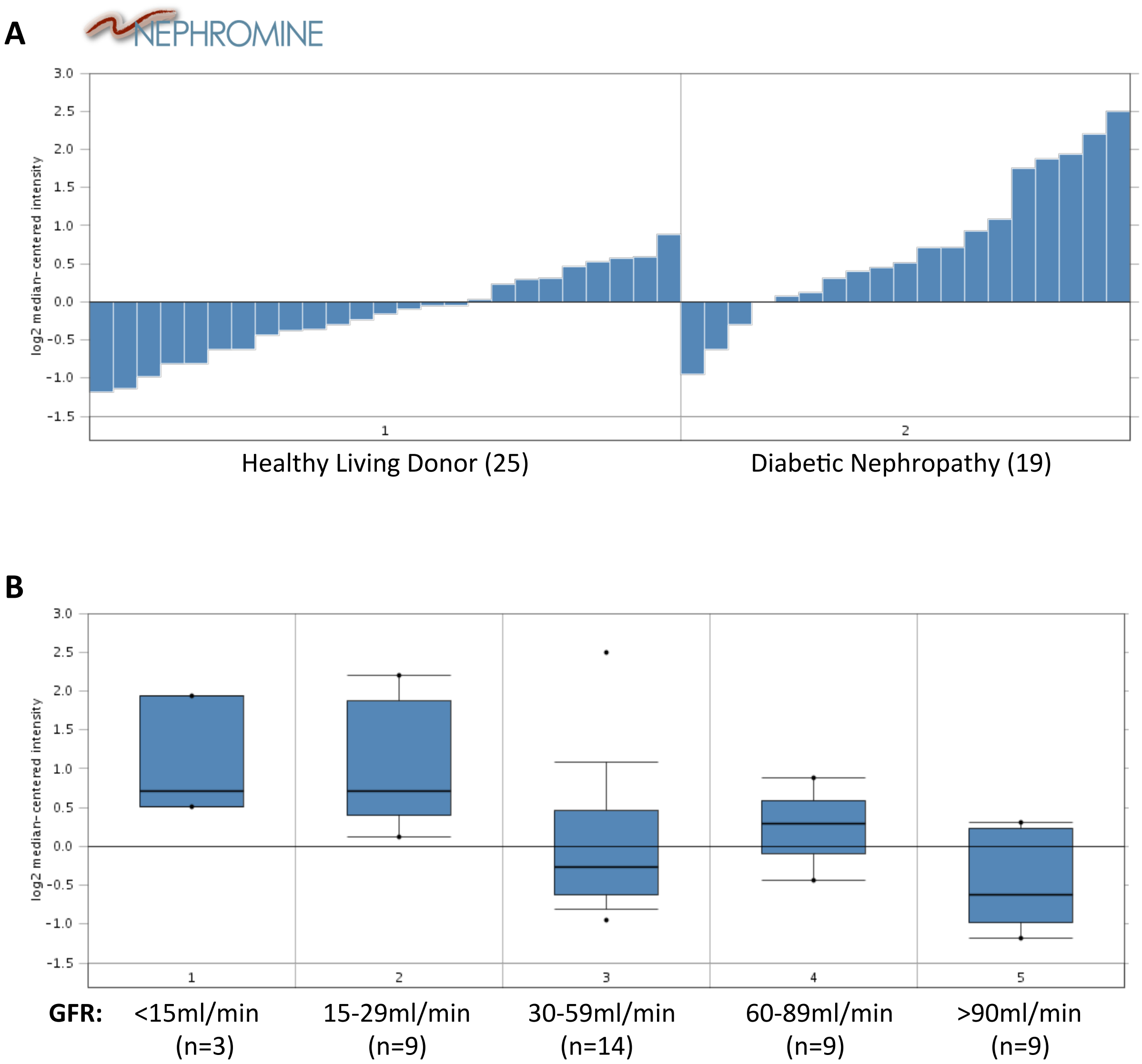

Supplementary Figure 2: Levels of *RTN1* mRNA expression are higher in kidneys of DN patients
Data from Nephromine.org: (A) Microarray studies of kidneys from patients with DN showing that *RTN1* mRNA levels are higher in DN as compared to normal kidneys. (B) *RTN1* mRNA expression inversely correlated with eGFR. Correlation between *RTN1* mRNA levels and eGFR was calculated by using the data of RTN1 mRNA levels and eGFR from individual patients. Pearson test was used (R=-0.56, P=7.8x10^{-5}, n=44).

Supplementary Figure 3

A

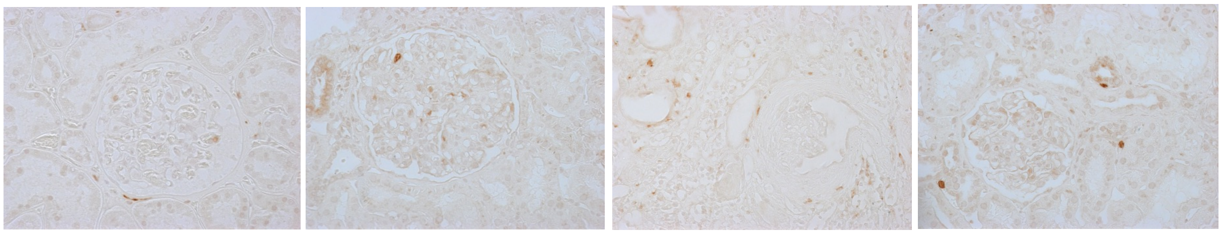

B

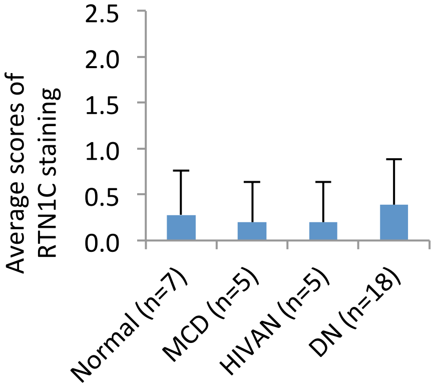

Supplementary Figure 3: RTN 1C expression in human kidney disease is not altered. (A) Representative immunostaining of RTN1C in kidney sections of patients with minimal change disease (MCD, n=5), HIV-associated nephropathy (HIVAN, n=5) and diabetic nephropathy (DN, n=18). Scale bar: 50µm. B) Semi-quantitative scoring of RTN1C staining.

Supplementary Figure 4

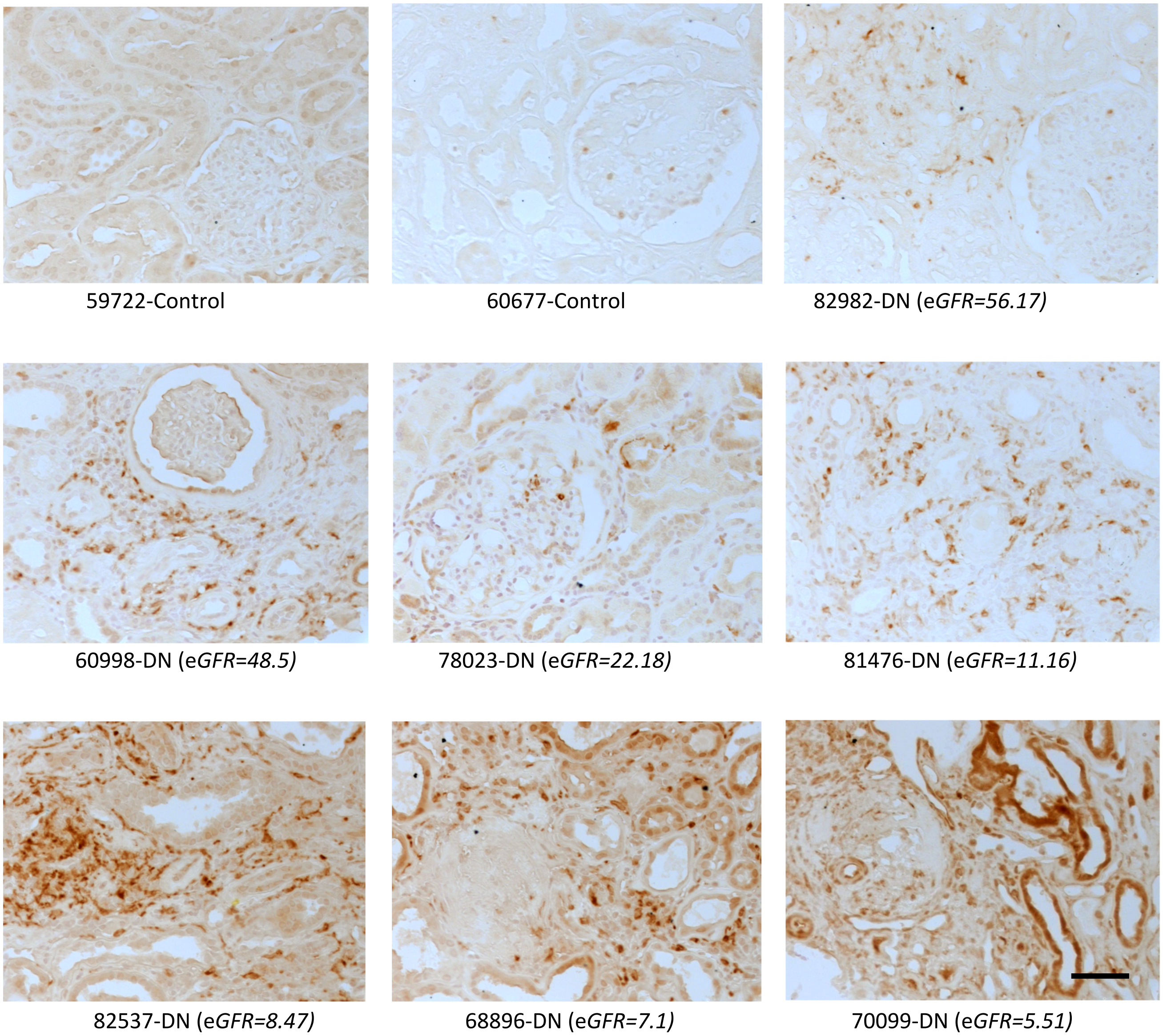

Supplementary Figure 4: Immunostaining of RTN1A in kidneys of patients with DN
Immunostaining of RTN1A was performed in kidneys from patients with DN (n=18) as compared to normal kidneys from nephrectomy samples (n=7) and the representative pictures are shown here with eGFR of corresponding patient shown in the parenthesis. Scale bar: 50μm. Immunostaining was done in duplicates.

Supplementary Figure 5

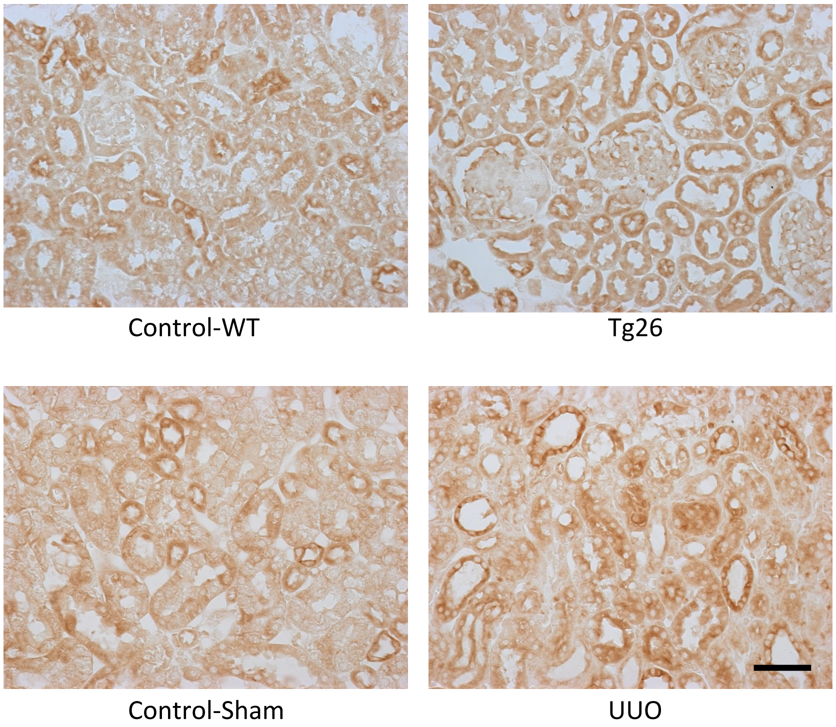

Supplementary Figure 5: RTN1C expression does not increase in murine models of HIVAN and renal fibrosis
Representative immunostaining of RTN1C in mouse kidney sections of controls, Tg26 and UUO. Immunostaining was done in duplicates. Scale bar: 50µm

Supplementary Figure 6

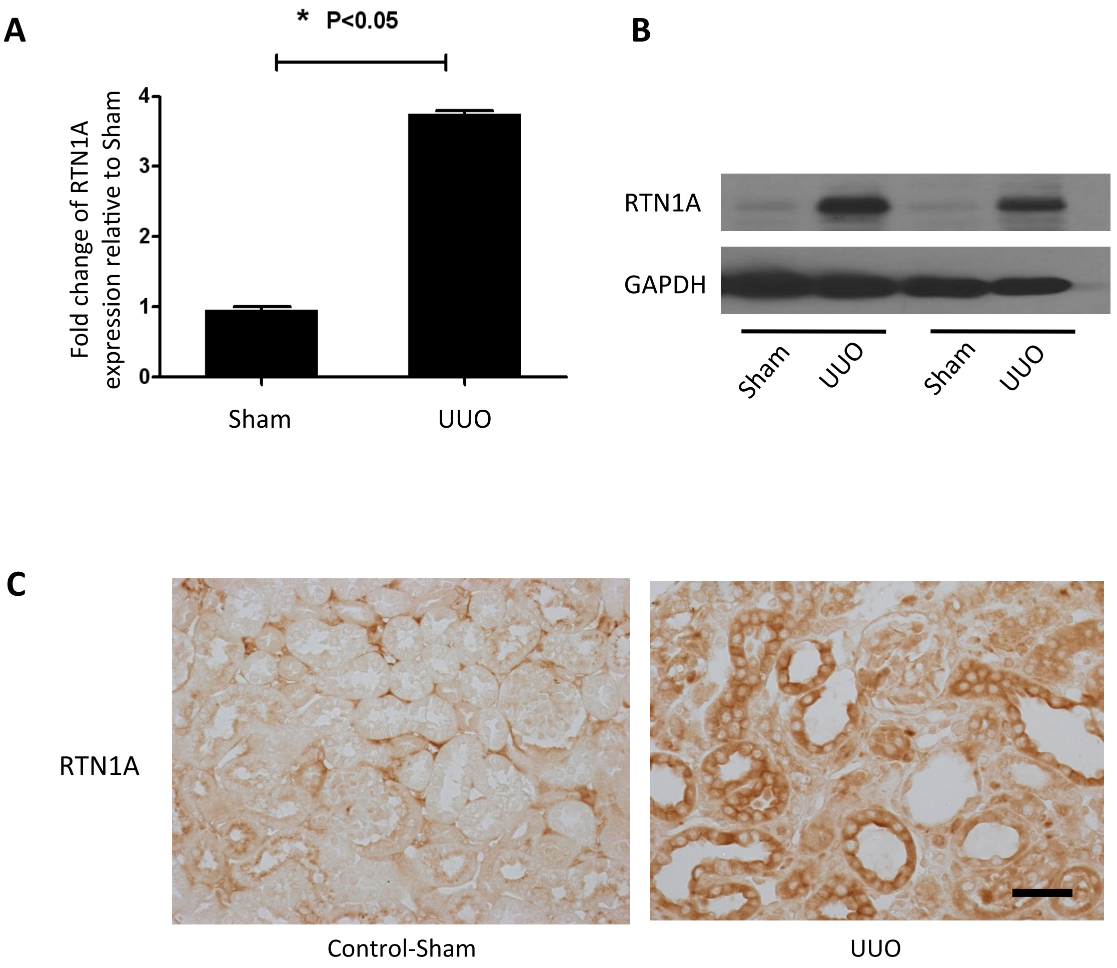

Supplementary Figure 6: *Rtn1a* expression is increased in kidneys of UUO mice
(A) *Rtn1a* mRNA levels in kidney cortices of sham operated (Sham) and UUO mice (7 days after UUO or sham procedure) as assessed by real-time PCR. n=3, $^{\#}P<0.05$, compared to sham-operated mice. (B) Western blot analysis of protein lysates from the kidney cortices for RTN1A. (C) Immunostaining of UUO and Sham kidneys. Scale bar: 50μm. Each PCR experiment was done in triplicate. Immunostaining and western blots were done in duplicate. The data were expressed as mean ± SD. The two-sided unpaired *t*-test was used.

Supplementary Figure 7

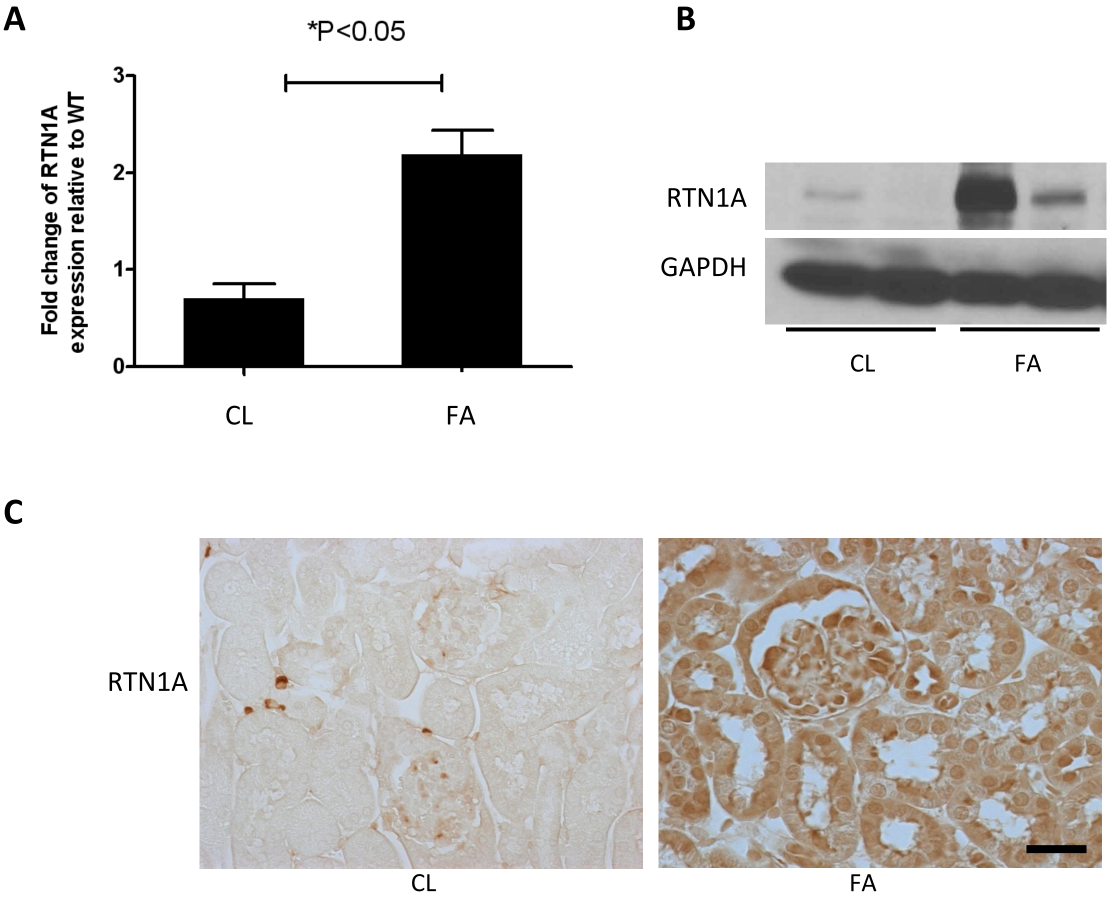

Supplementary Figure 7: *Rtn1a* expression is increased in kidneys of mice with folic-acid-induced nephropathy

(A) *Rtn1a* mRNA levels in kidney cortices of mice with folic acid-induced nephropathy (FA) and control mice were assessed by real time PCR. n=3, $^{\#}P<0.05$, compared to control group (CL). (B) Western blot analysis of kidney lysates for RTN1A. (C) Immunohistochemistry staining of RTN1A in kidney sections of CL and FA. Scale bar: 50µm. Each PCR experiment was done in triplicate. Immunostaining and western blots were done in duplicate. The data were expressed as mean ± SD. The two-sided unpaired *t*-test was used.

Supplementary Figure 8

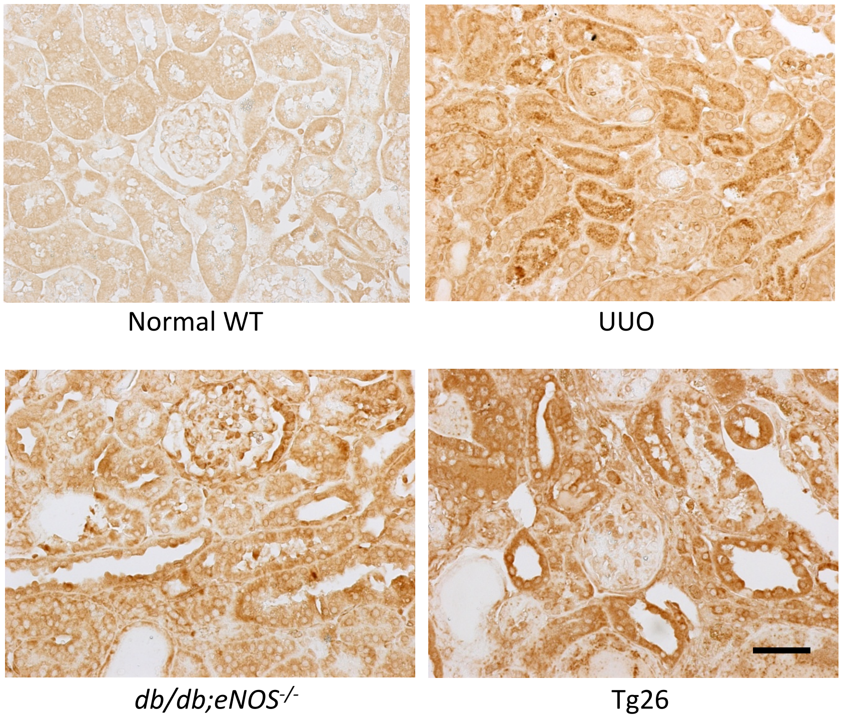

Supplementary Figure 8: Immunostaining of RTN1A in the kidneys of multiple animal models of kidney disease using a different antibody

Immunostaining was performed as above in the kidney sections from normal, UUO, *db/db*, and Tg26 mice using a rabbit polyclonal antibody against mouse RTN1A from a different company (CD Creative Diagnostics, Cat# DPABT-H23572). The representative pictures of three different mice are shown.

Supplementary Figure 9

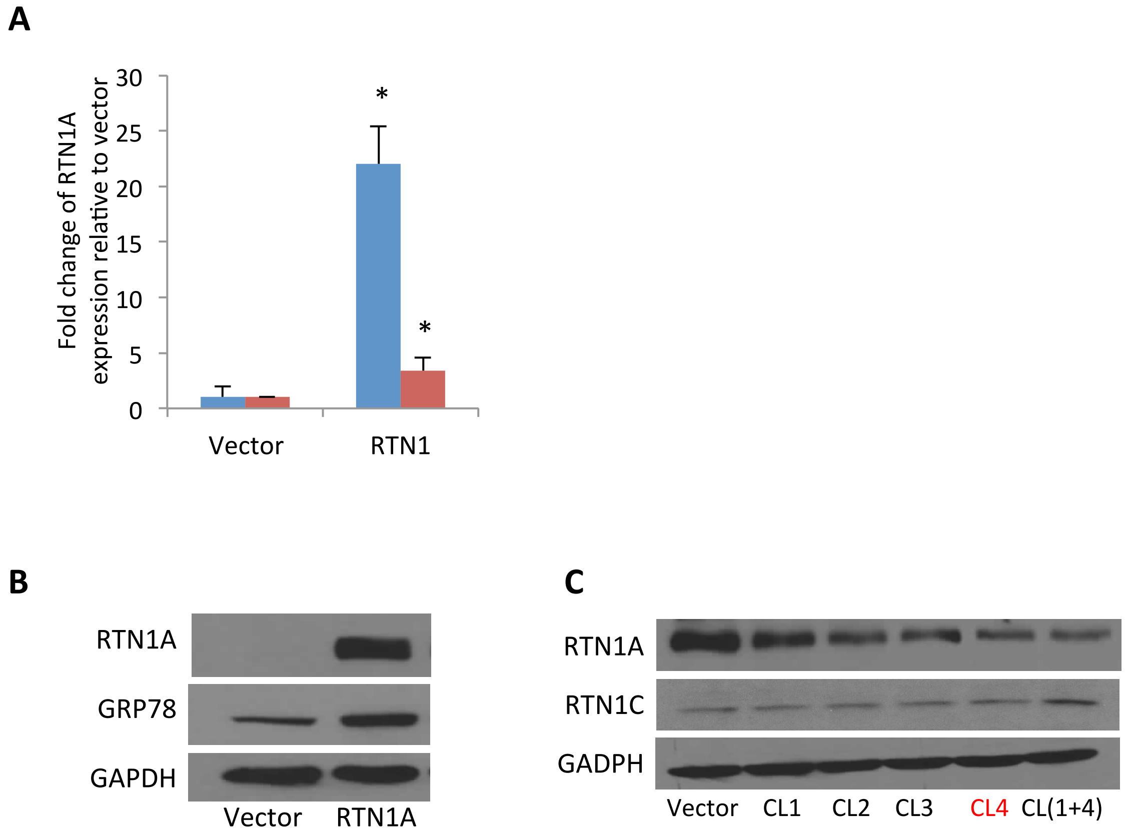

Supplementary Figure 9: *RTN1A* over-expression in 293T cells
(A) The mRNA levels of *RTN1A* and *GRP78* were assessed in 293T cells transfected with an *RTN1A* expression construct by real time PCR. n=3, $^{*}P<0.05$ compared to cells transfected with the empty vector (Vector). (B) Western blot analysis was also performed in these cells for RTN1A and GRP78. (C) Western blots of RTN1A and RTN1C in 293T cells transfected with scrambled shRNA (Vector) or specific shRNA lentivector targeting *RTN1A* (clones CL-1, -2, -3, -4 or a mix of CL-1 and CL-4 [1+4]) for knockdown. Each PCR experiment was done in triplicate. Western blot was done in duplicate. The data were expressed as mean ± SD. The two-sided unpaired *t*-test was used.

Supplementary Figure 10

A

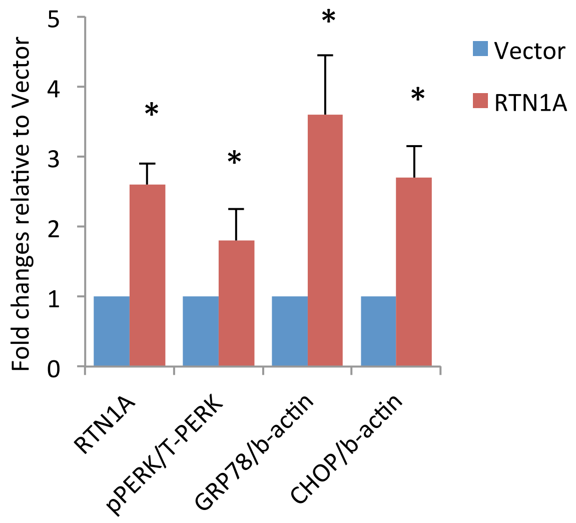

B

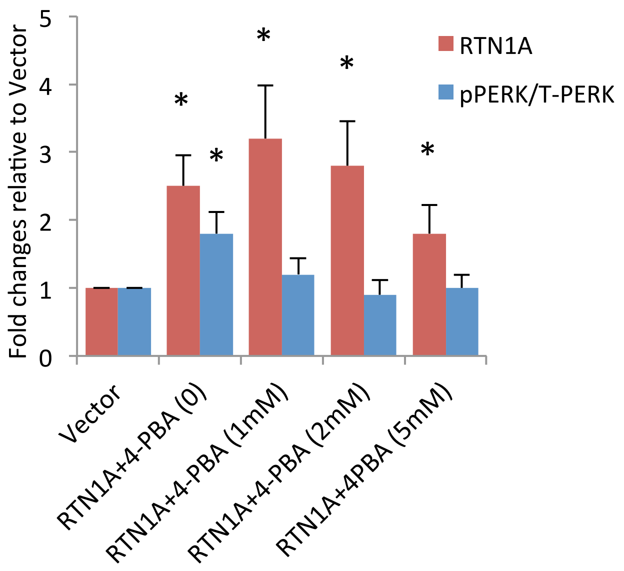

C

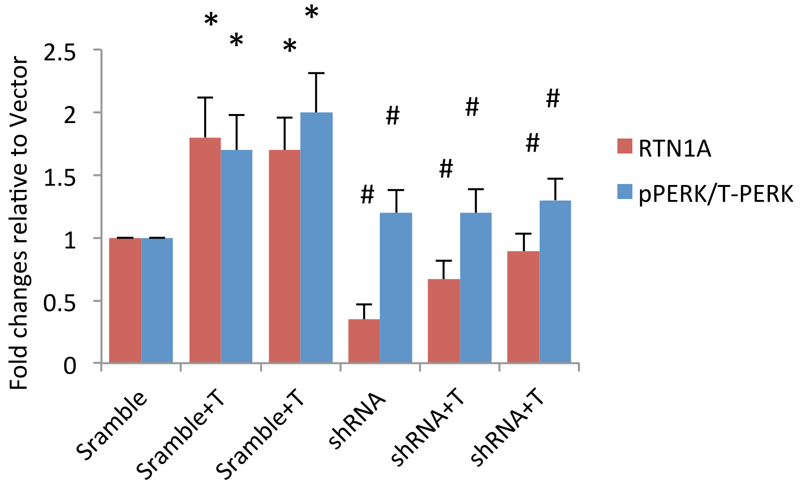

Supplementary Figure 10: Densitometry analysis of the western blots shown in the Figure 2A-C

The western blots of the results shown in Figure 2A-2C were analyzed by densitometry and the corresponding data are shown in the Supplementary Figure 8A to 8C. **P*<0.05 when compared to cells transfected with control vector; #*P*<0.05 when compared to cells transfected with scramble shRNA and treated with tunicamycin (T), n=3. The data were expressed as mean ± SD. The two-sided unpaired *t*-test was used.

Supplementary Figure 11

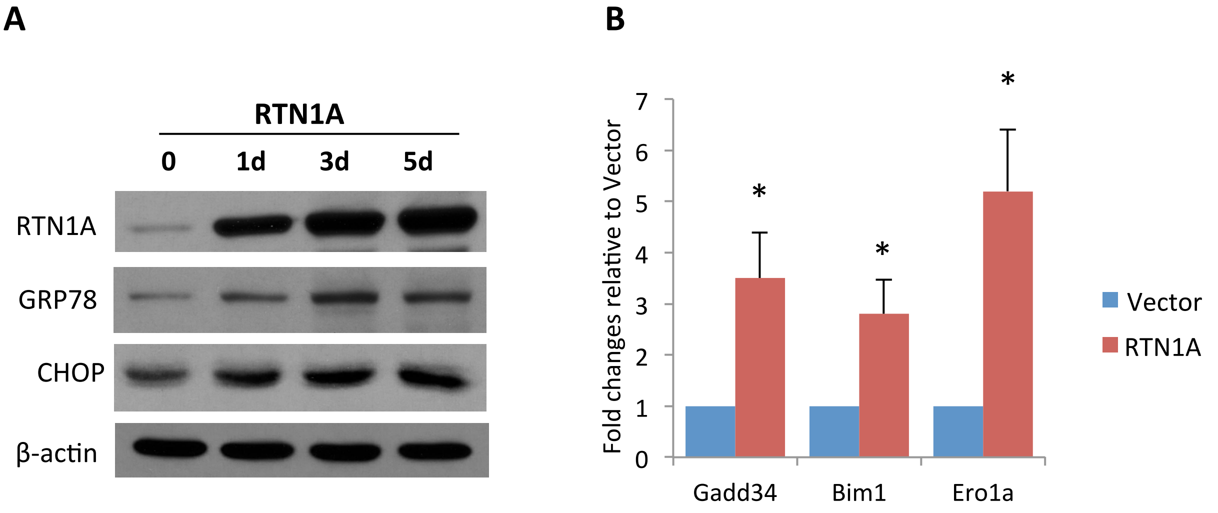

Supplementary Figure 11: Time course of RTN1A overexpression-induced ER stress and expression of CHOP target genes in HK2 cells

HK2 cells were transfected with *RTN1A* for 1, 3, 5 days and the expression of RTN1A and ER stress markers (GRP78 and CHOP) was analyzed by western blots. (B) The known target genes of CHOP (Gadd34, Bim1, and Ero1a) were analyzed by real-time PCR in HK2 cells with overexpression of RTN1A for 5 days. *P<0.01 when compared to cells transfected with control vector, n=3. Western blot was done in duplicate. The data were expressed as mean ± SD. The two-sided unpaired t-test was used.

Supplementary Figure 12

A

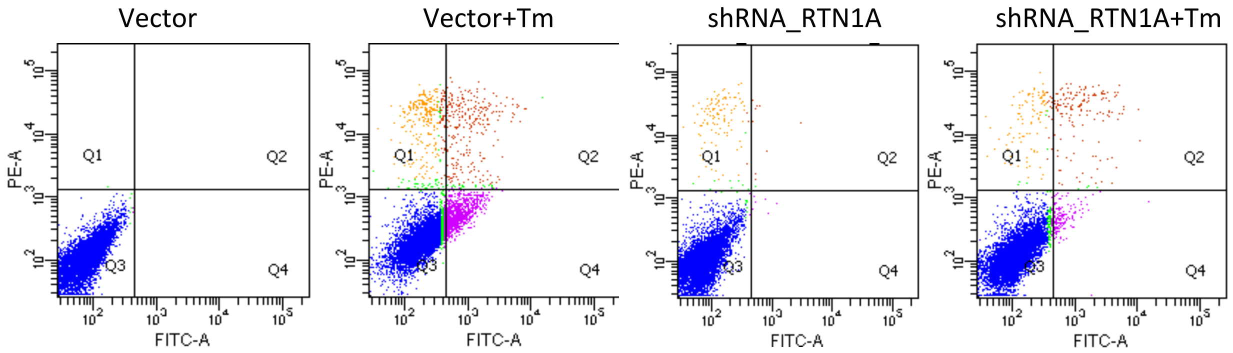

B

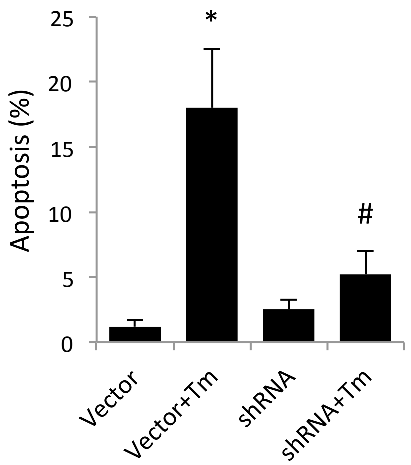

Supplementary Figure 12: Knoc down of RTN1 attenuates Tunicamycin-induced apoptosis of HK2 cells
(A) Flow cytometric analysis of Annexin V and propidium Iodide double-labeled HK2 cells. HK2 cells were infected with a lentiventor containing scrambled shRNA (vector) or an *RTN1A*-specific shRNA (shRNA) 5 days prior to the apoptosis study. Infected cells were treated with 25ng/ml of Tunicamycin (Tm) or DMSO as control for 72hrs after transfection. (B) Summary of apoptosis data on HK2 cells. *P<0.001 compared to vector, #P<0.01 compared to vector+Tm, n=3. Each experiment of Flow cytometry was done in duplicate. Western blot was done in duplicate. The data were expressed as mean ± SD. The two-sided unpaired *t*-test was used.

Supplementary Figure 13

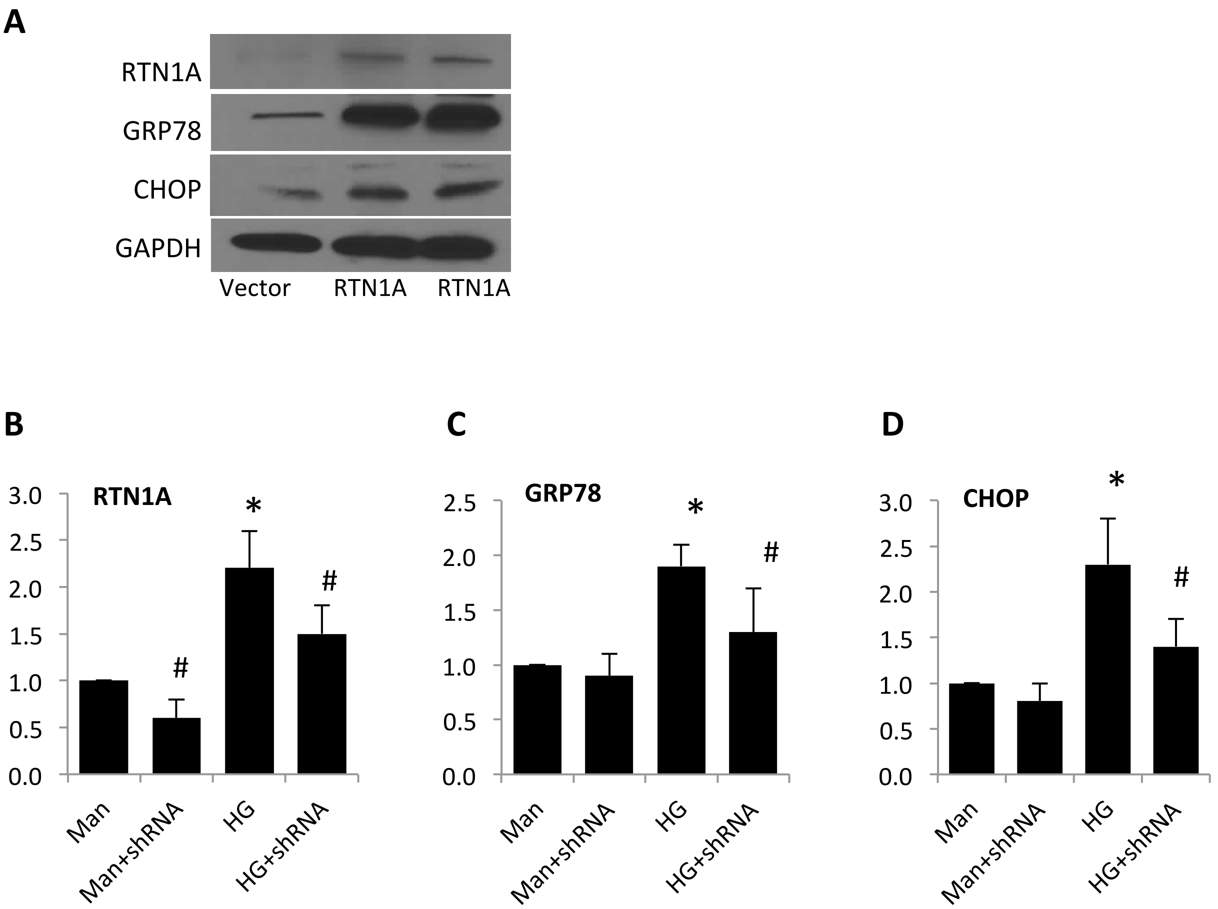

Supplementary Figure 13: RTN-1A mediates high glucose-induced ER stress in cultured human podocytes.

(A) Human podocytes were cultured as described and transfected with either control vector or *RTN1A* for 3 days and western blot analysis was performed for RTN1A, GRP78, CHOP, and GAPDH. (B-D) Podocytes were infected with lentiviral vectors containing shRNA for RTN1A for 3 days and then cells were further incubated with either normal glucose (6 mM glucose and 24 mM mannitol for equal osmolarity) or high glucose (30 mM) for 2 days. Real-time PCR was performed in these cells for *RTN1A* (B), *GRP78* (C), and *CHOP* (D). **P*<0.01 compared to cells incubated with mannitol, #*P*<0.01 compared to cells incubated wit high glucose, n=3. Each PCR experiment was done in triplicate and western blot was done in duplicate. The data were expressed as mean ± SD. The ANOVA with Bonferroni correction was used.

Supplementary Figure 14

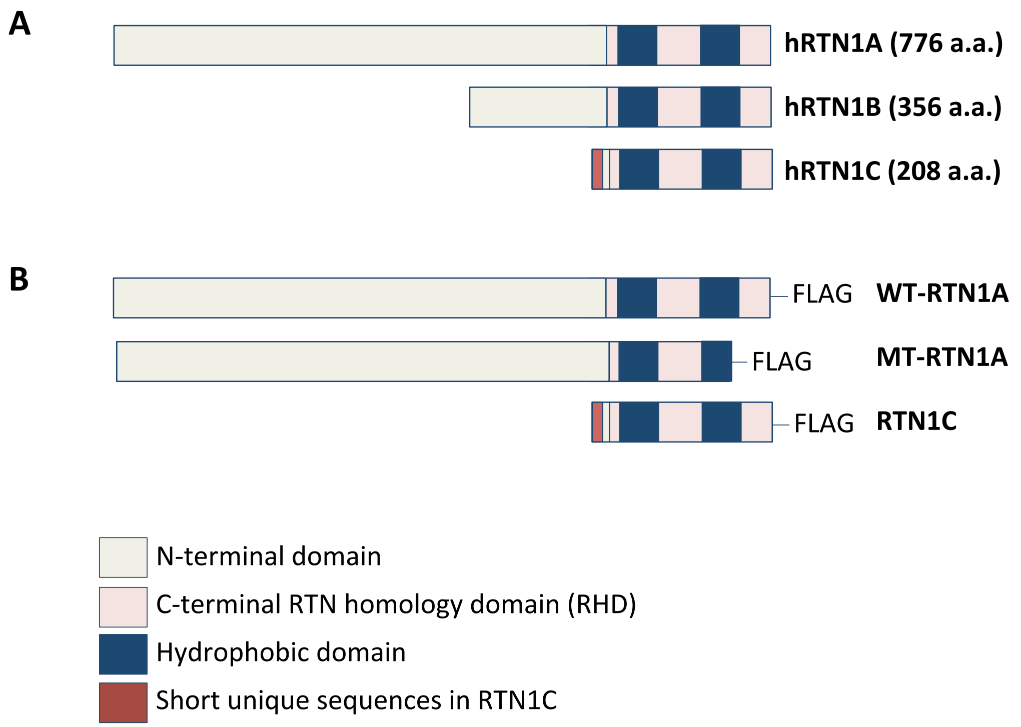

Supplementary Figure 14: Schematic representation of RTN1 isoforms and RTN1 overexpression constructs.
(A) RTN1 isoforms vary in the length of the hydrophilic N-terminal domain, but all share the same C-terminal reticulon homology domain (RHD). RHD is characterized by two long hydrophobic domains separated by a short hydrophilic loop that is followed by a short C-terminal tail. (B) Schema of FLAG-tagged RTN1 overexpression constructs used.

Supplementary Figure 15

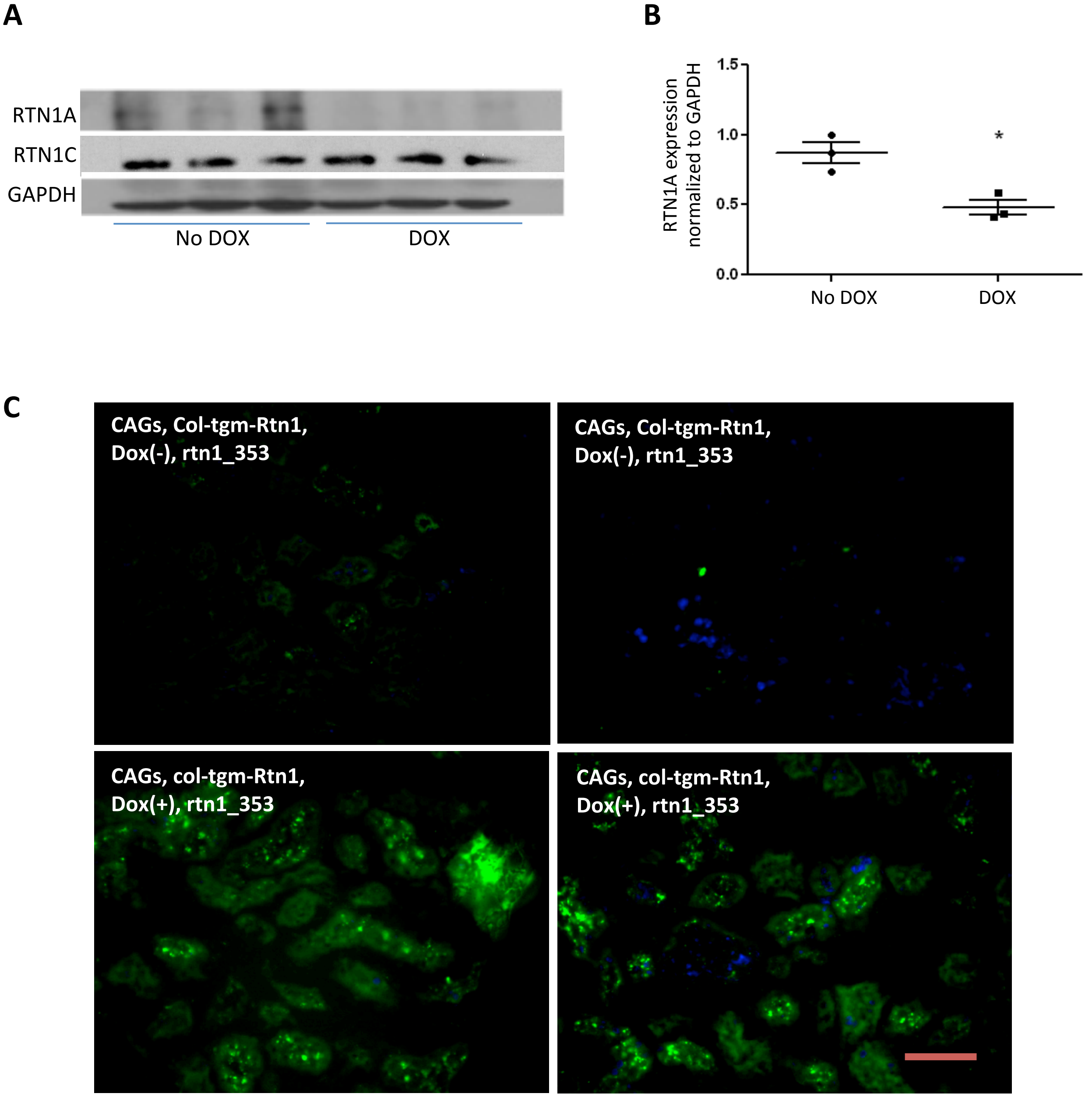

Supplementary Figure 15: *CAGs-rtTA;Rtn1^{siRNA}* mice (line *rtn1_353)*

CAGs-rtTA;Rtn1^{siRNA} mice were generated by crossing CAGs-rtTA mice with RTN1^{siRNA} mice. Mice were fed with DOX at (600mg/kg) twice a day for 3 weeks from age of 6 weeks to 9 weeks. Mice were sacrificed and kidney cortices were used for western blot analysis of RTN1A and RTN1C (A) and real-time PCR analysis of *Rtn1a* mRNA levels (B) in *CAGs-rtTA;Rtn1^{siRNA}* mice fed with or without DOX. GFP expression was also examined in the kidney of these mice (C). *P<0.05, n=3. Each PCR experiment was done in triplicate. Western blot was done in duplicate. The data were expressed as mean ± SD. The two-sided unpaired *t*-test was used.

Supplementary Figure 16

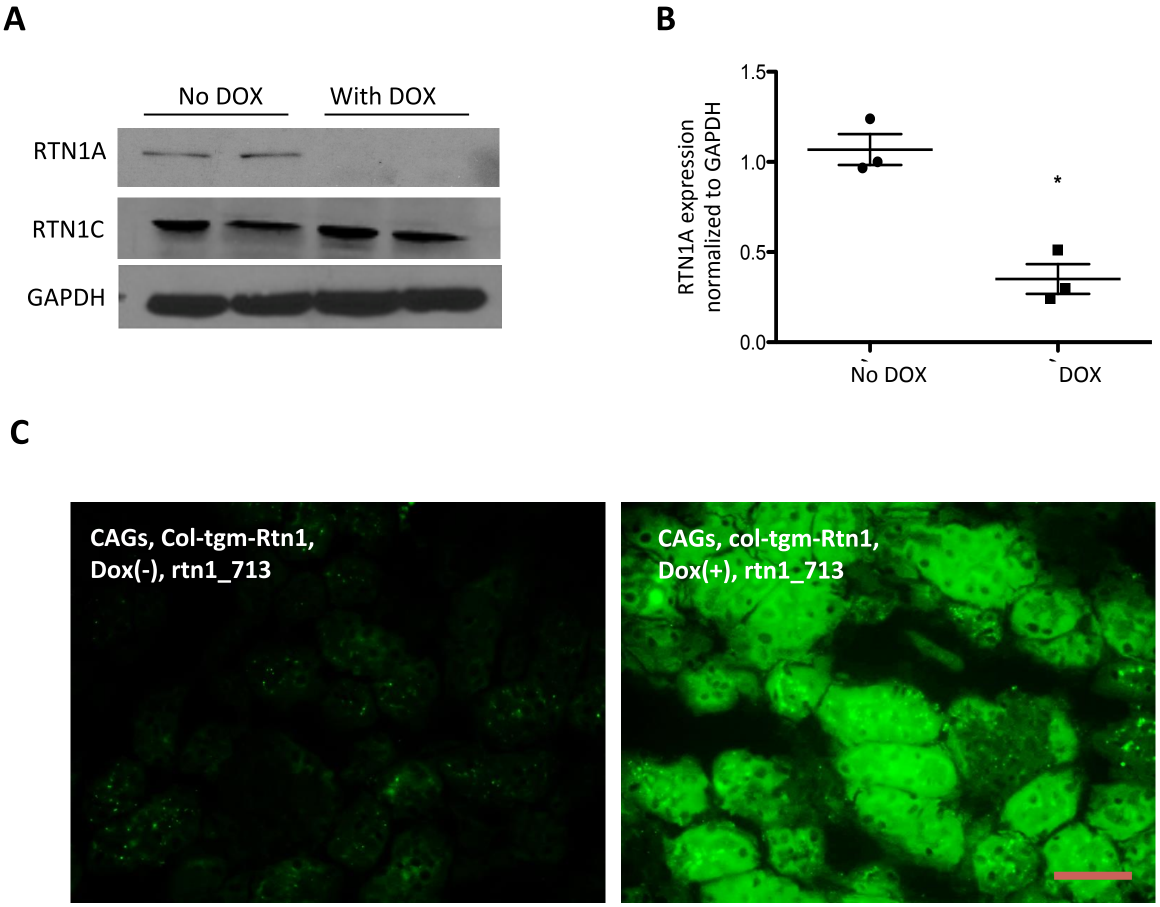

Supplementary Figure 16: *CAGs-rtTA;Rtn1^{siRNA}* mice (line *rtn1_713).*
CAGs-rtTA;Rtn1^{siRNA} mice were generated by crossing CAGs-rtTA mice with RTN1^{siRNA} mice. Mice were fed with DOX at (600mg/kg) twice a day for 3 weeks from age of 6 weeks to 9 weeks. Mice were sacrificed and kidney cortices were used for western blot analysis of RTN1A and RTN1C (A) and real-time PCR analysis of *Rtn1a* mRNA levels (B) in *CAGs-rtTA;Rtn1^{siRNA}* mice fed with or without DOX. GFP expression was also examined in the kidney of these mice (C). *P<0.05, n=3. Each PCR experiment was done in triplicate. Western blot was done in duplicate. The data were expressed as mean ± SD. The two-sided unpaired t-test was used.

Supplementary Figure 17

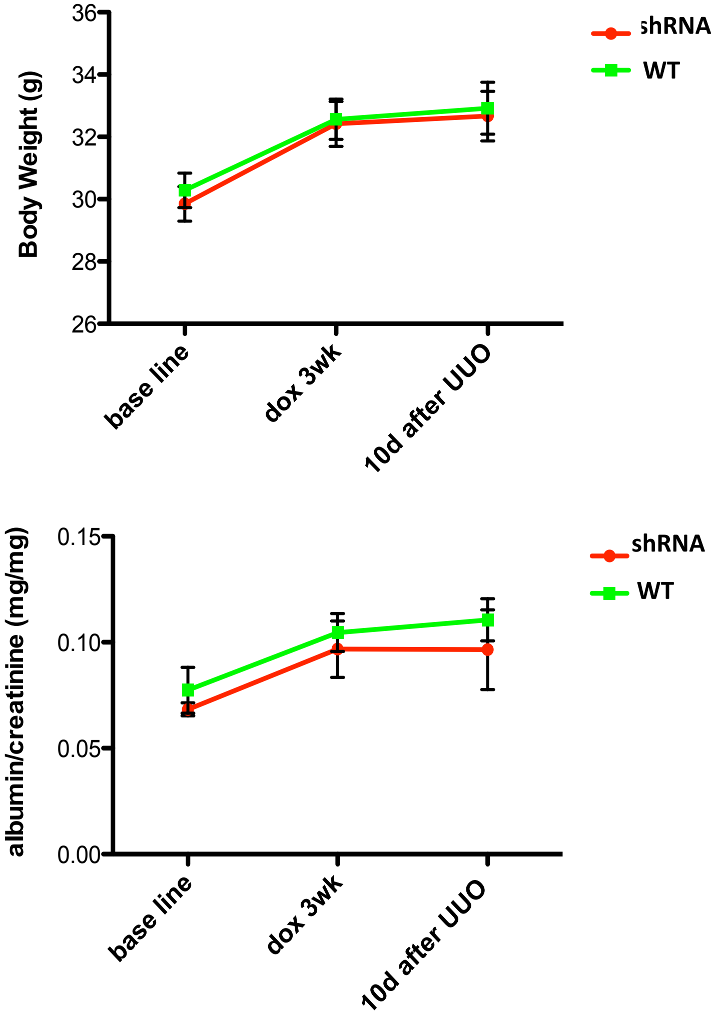

Supplementary Figure 17: Baseline data of *CAGs;Rtn1*^{siRNA} and *CAGs;Luc*^{siRNA} mice after UUO.
Body weight (top) and urine albumin/creatinine (bottom) were measured in both *CAGs;Rtn1*^{siRNA} (shRNA) and *CAGs;Luc*^{siRNA} (WT) mice prior to feeding of DOX, the day of surgery, and 10 days after UUO. There were no differences between the two groups, n=6.

Supplementary Figure 18

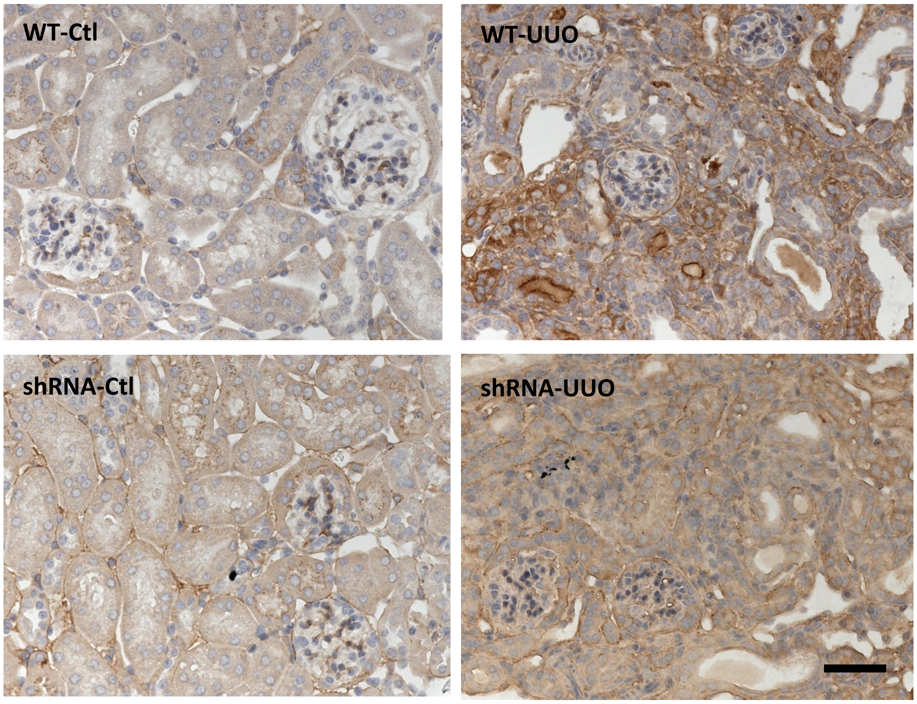

Supplementary Figure 18: Rtn1-a expression in both shRNA and WT mice with or without UUO. RTN1A protein levels were determined by immunostaining in kidneys of both *CAGs;Rtn1*^{siRNA} (shRNA) and *CAGs;Luc*^{siRNA} (WT) mice with sham-operation (Ctl) or UUO. Immunostaining was done in duplicate. The representative pictures of three mice are shown. Scale bar: 50µm

Supplementary Figure 19

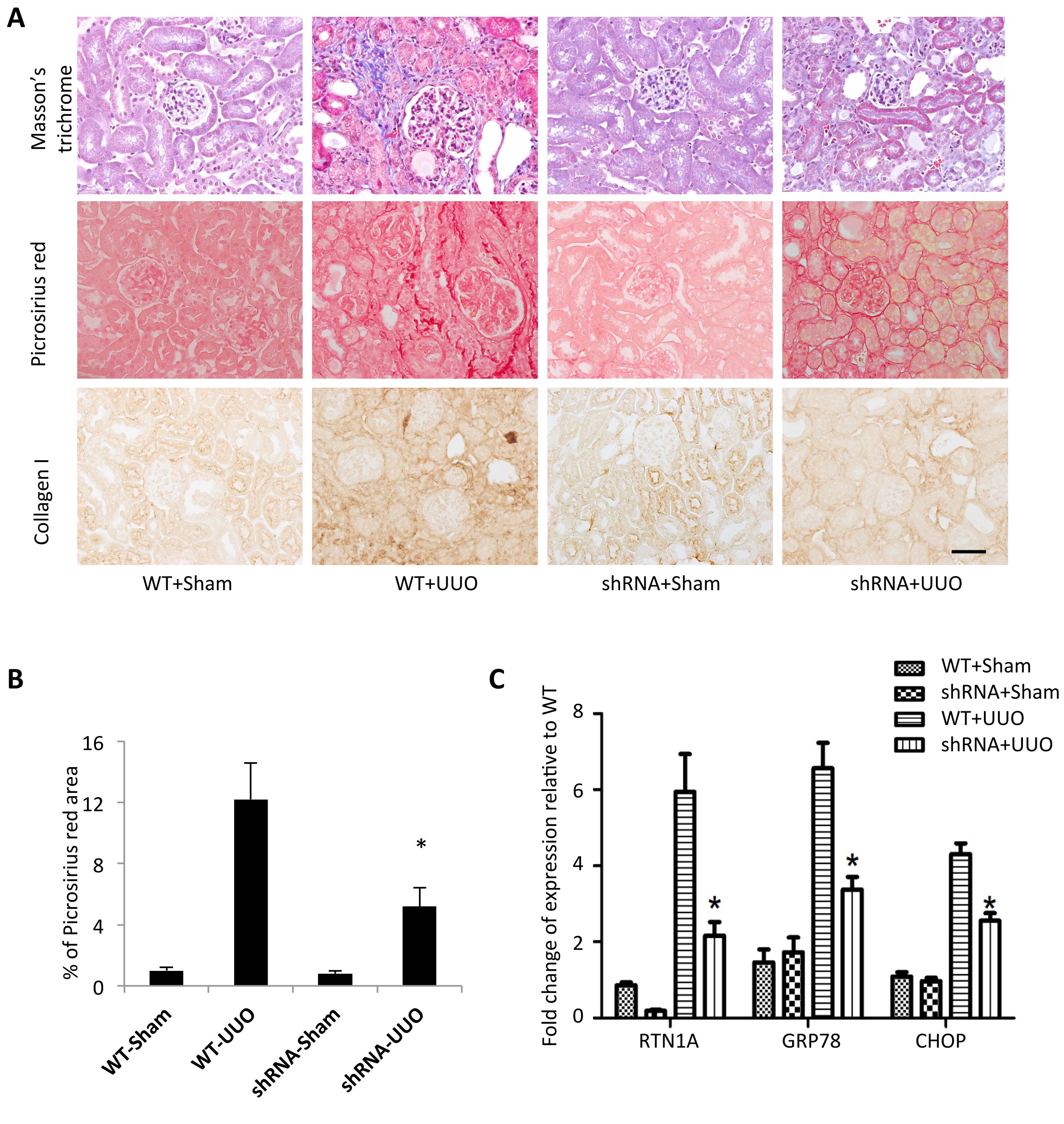

Supplementary Figure 19: Validation of the effect of Rtn1a knockdown in the UUO mice using a second line of *CAGs rtTA;RTN1^{RNAi}* mice.

UUO was created in the 2nd line of *Rtn1a* knockdown mice (shRNA) and the control mice (WT). The sham-operated mice were used as the controls. The mice were sacrificed 10 days post-UUO and the kidneys were removed for histology analysis (A). Scale bar: 50µm. The fibrosis score was determined by quantification of Picrosirius red staining (B). Total RNA was isolated from these kidneys for real-time PCR analysis of ER stress markers (C). *P<0.01 compared to WT-UUO mice, n=6. Immunostaining was done in duplicate. The representative pictures of six mice are shown. The data were expressed as mean ± SD. The ANOVA with Bonferroni correction was used.

Supplementary Figure 20

Figure 2B:

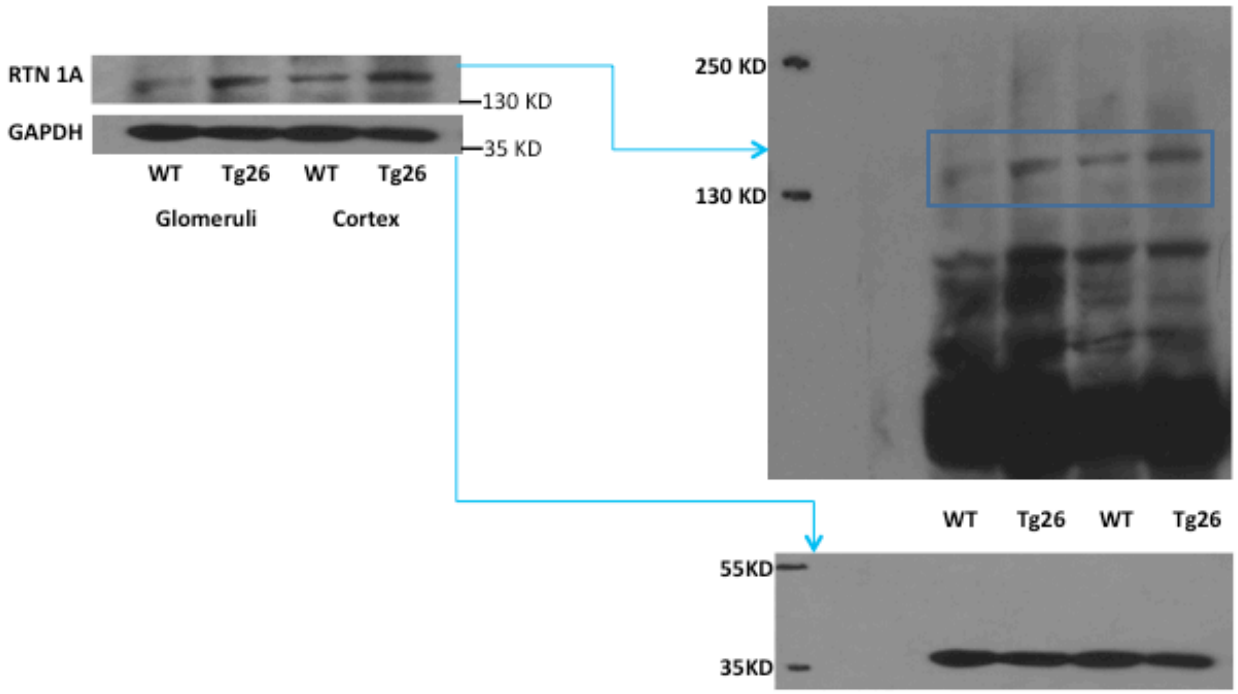

Figure 3A:

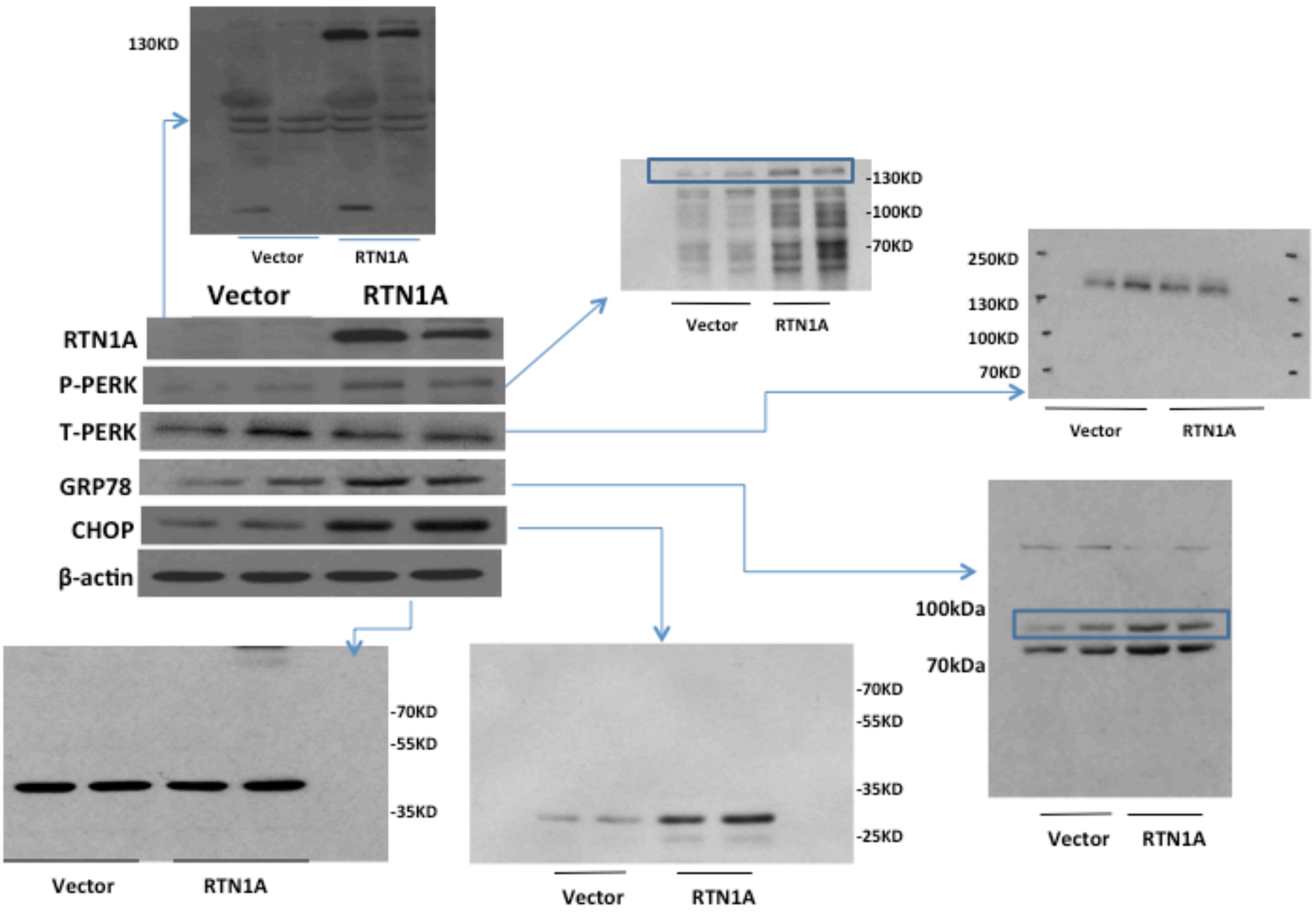

130KD
Vector
RTN1A
Vector
RTN1A
RTN1A
P-PERK
T-PERK
GRP78
CHOP
β-actin
-130KD
-100KD
-70KD
Vector
RTN1A
250KD
130KD
100KD
70KD
Vector
RTN1A
100kDa
70kDa
Vector
RTN1A
-70KD
-55KD
-35KD
Vector
RTN1A
-70KD
-55KD
-35KD
-25KD
Vector
RTN1A

Figure 3B.

Same blot in figure 4C was used

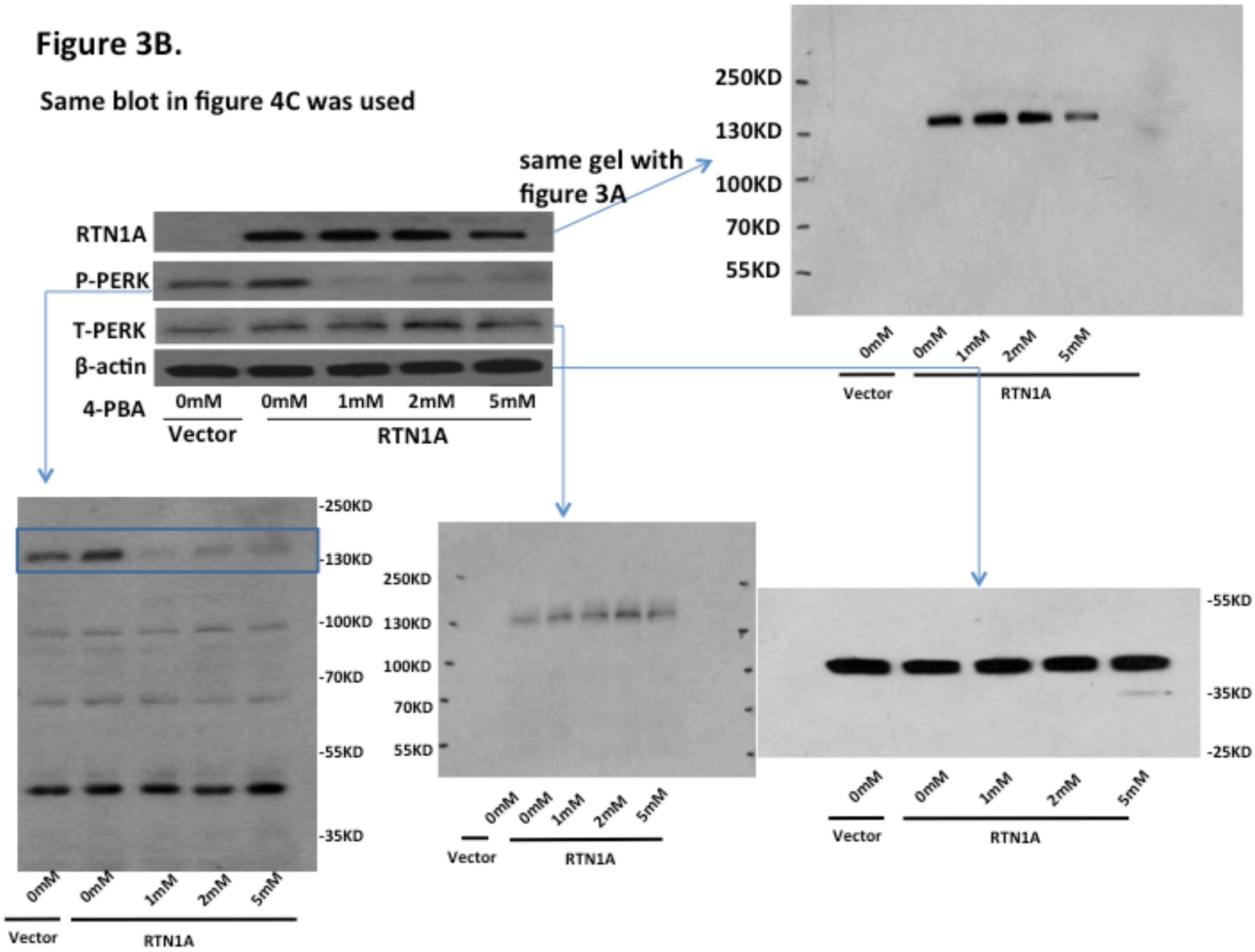

Figure 3C.

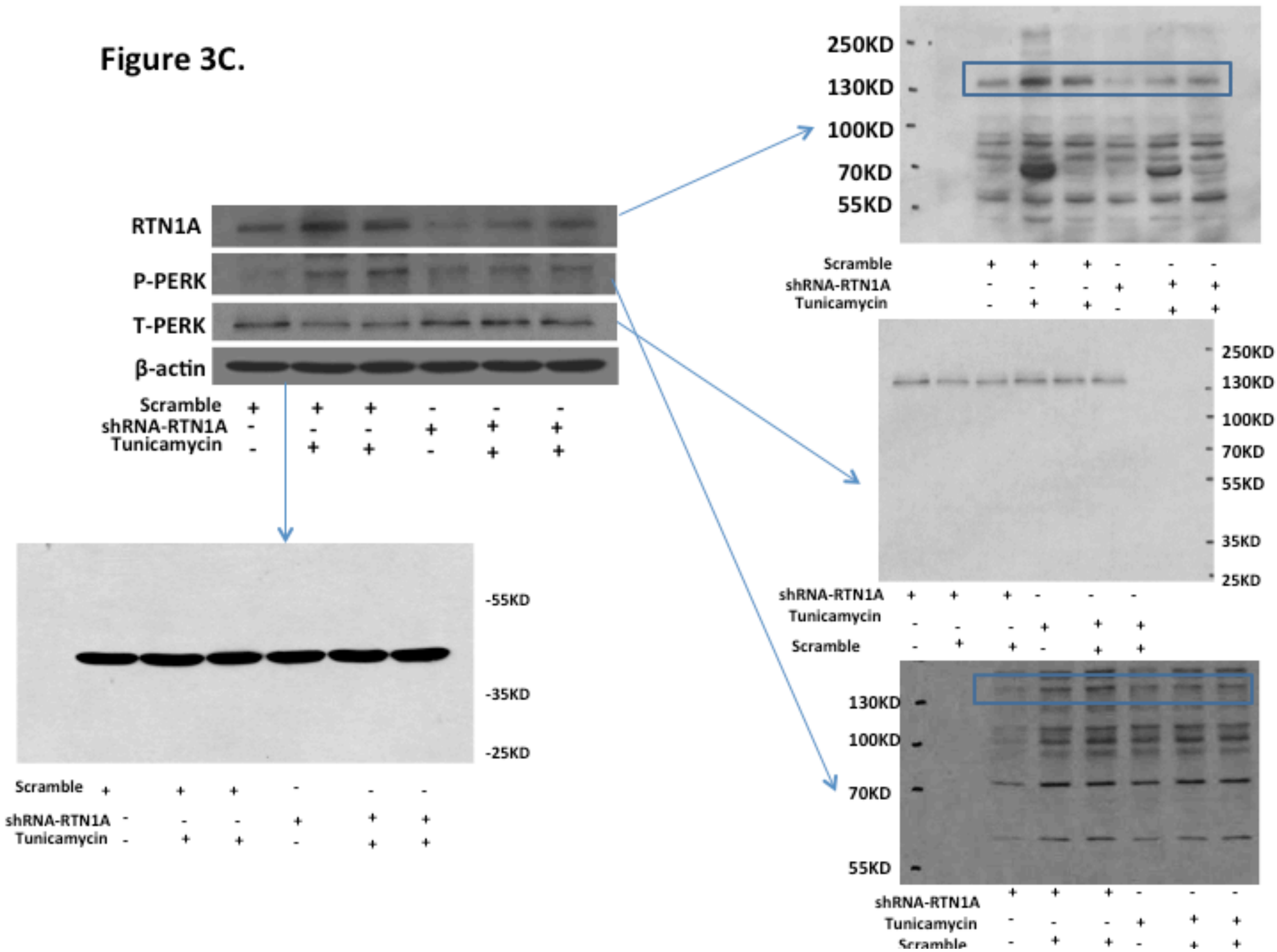

RTN1A
P-PERK
T-PERK
β-actin
Scramble
shRNA-RTN1A
Tunicamycin
250KD
130KD
100KD
70KD
55KD
35KD
25KD
-55KD
-35KD
-25KD

Figure 4C

Same blot in figure 3B was used

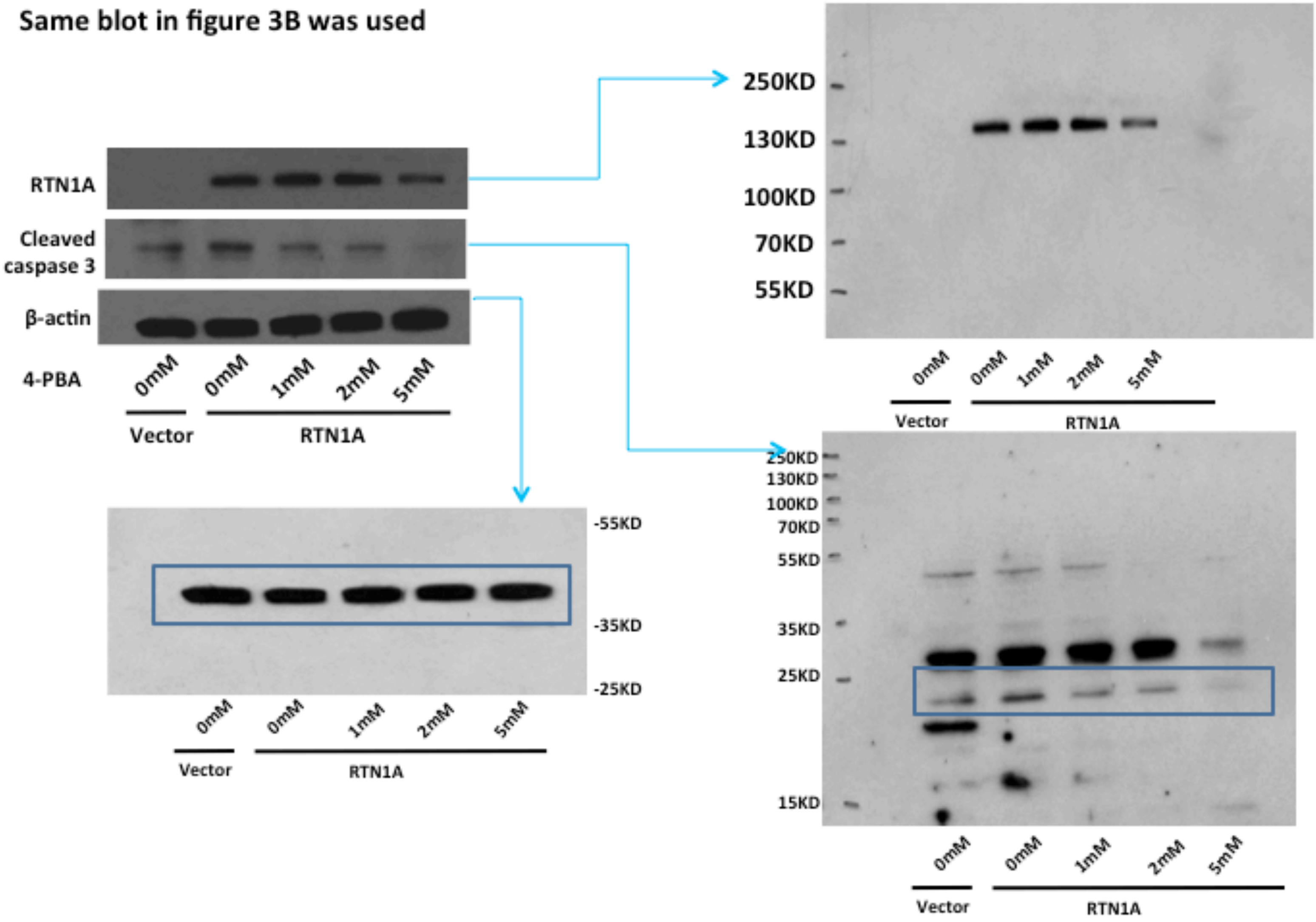

Figure 4F

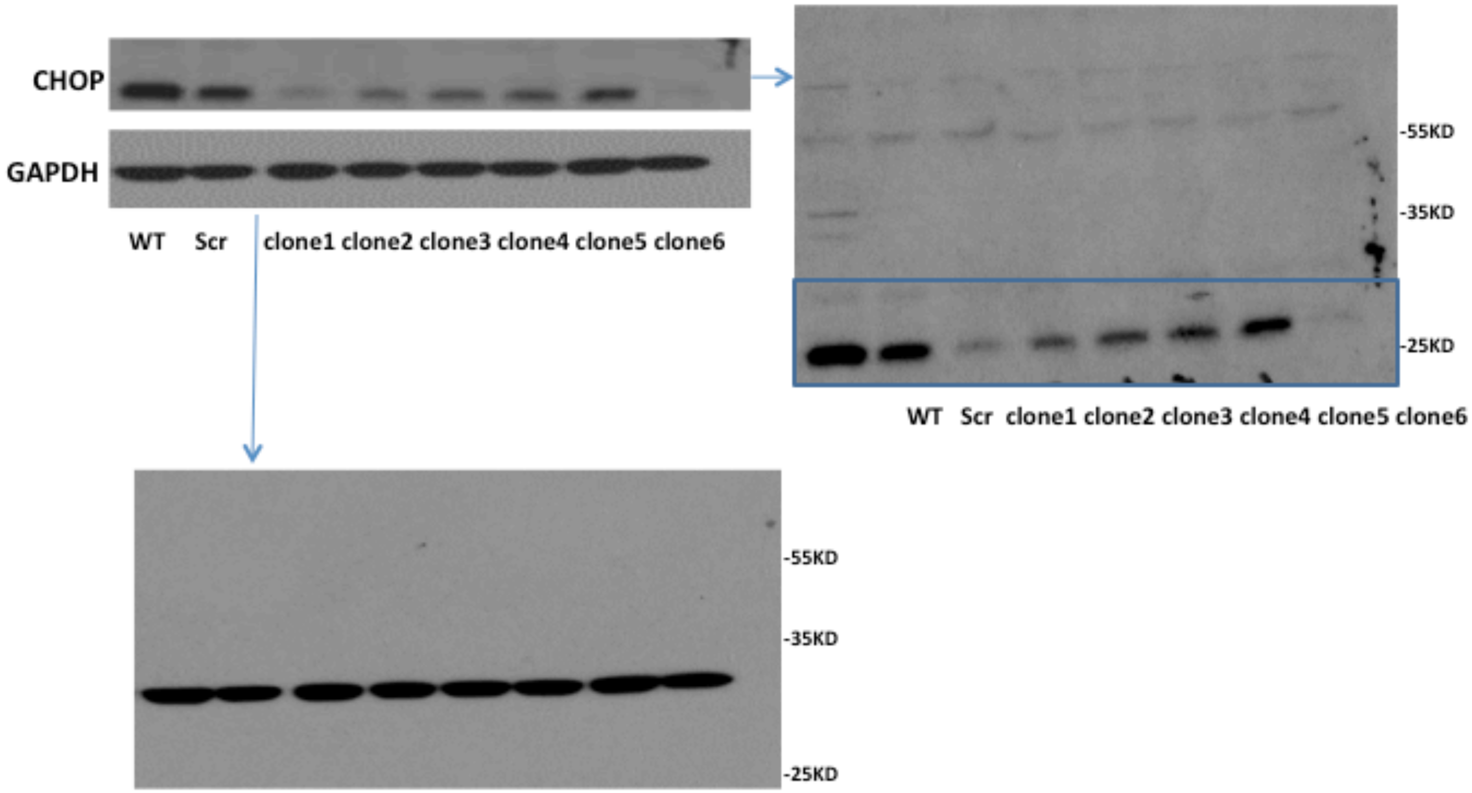
CHOP
GAPDH
WT Scr clone1 clone2 clone3 clone4 clone5 clone6
-55KD
-35KD
-25KD
WT Scr clone1 clone2 clone3 clone4 clone5 clone6
-55KD
-35KD
-25KD

Figure 4G.

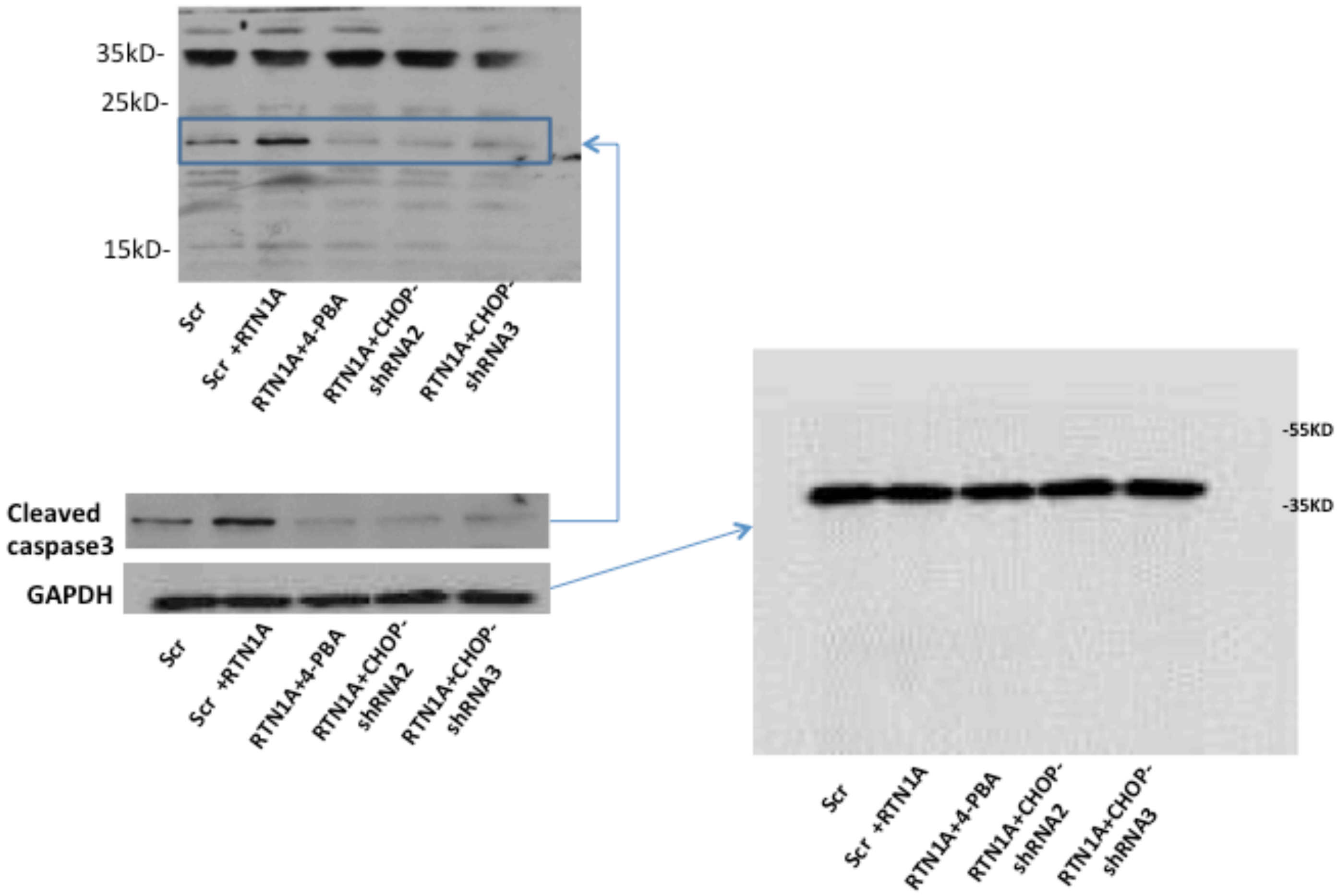

35kD-
25kD-
15kD-
Scr
Scr +RTN1A
RTN1A+4-PBA
RTN1A+CHOP-shRNA2
RTN1A+CHOP-shRNA3
Cleaved caspase3
GAPDH
Scr
Scr +RTN1A
RTN1A+4-PBA
RTN1A+CHOP-shRNA2
RTN1A+CHOP-shRNA3
-55KD
-35KD
Scr
Scr +RTN1A
RTN1A+4-PBA
RTN1A+CHOP-shRNA2
RTN1A+CHOP-shRNA3

Figure 5A

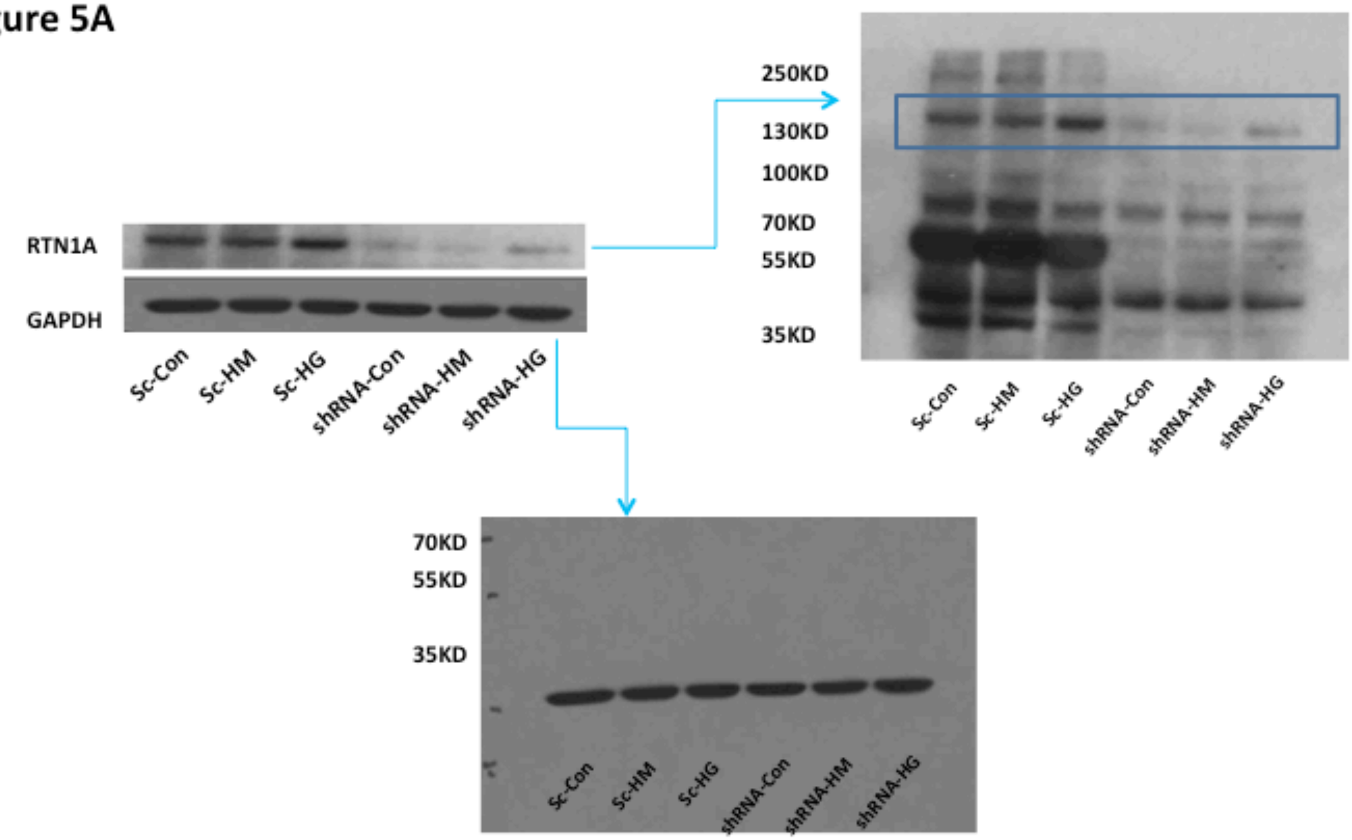

Figure 5I

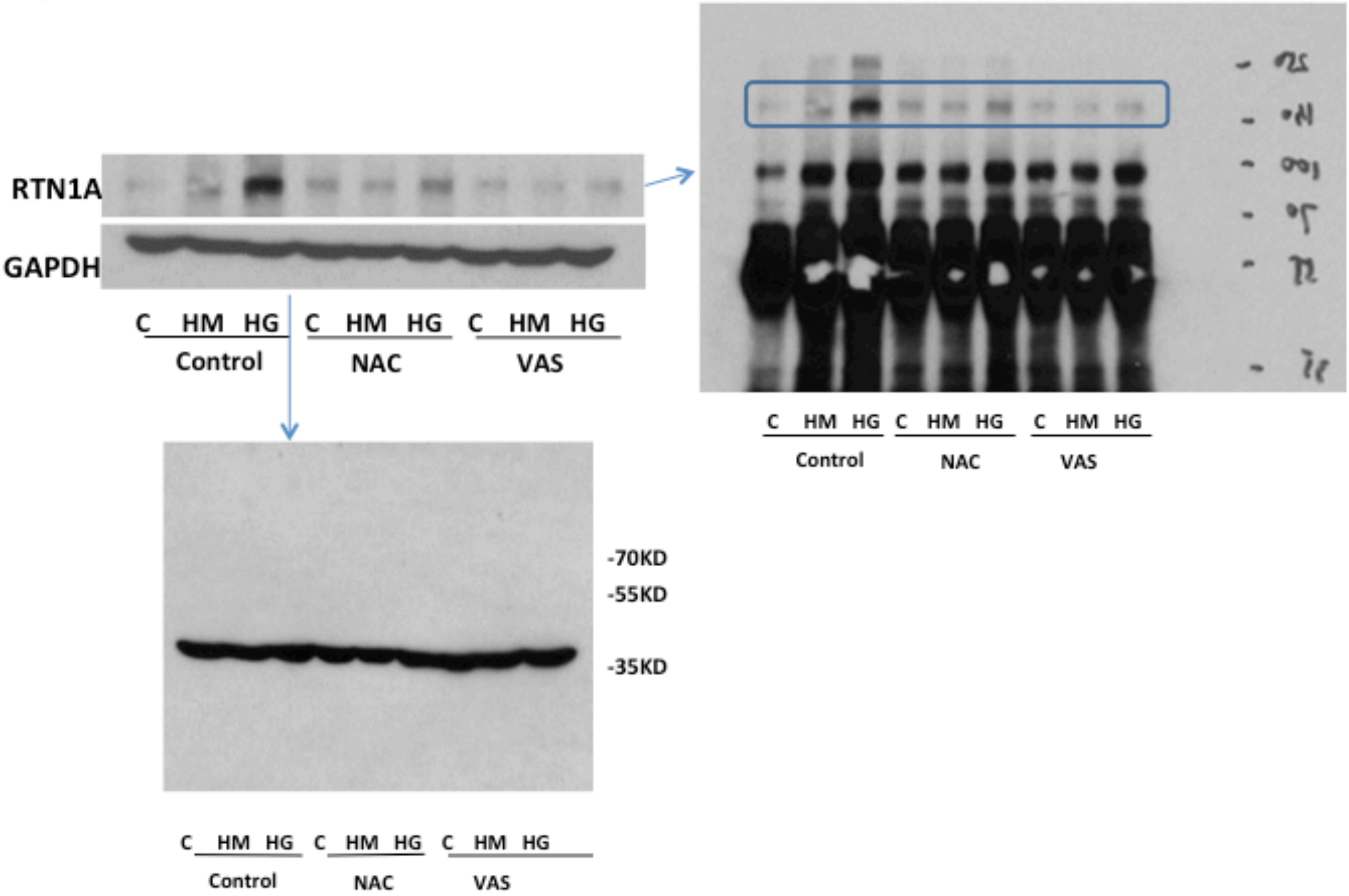

Figure 6E

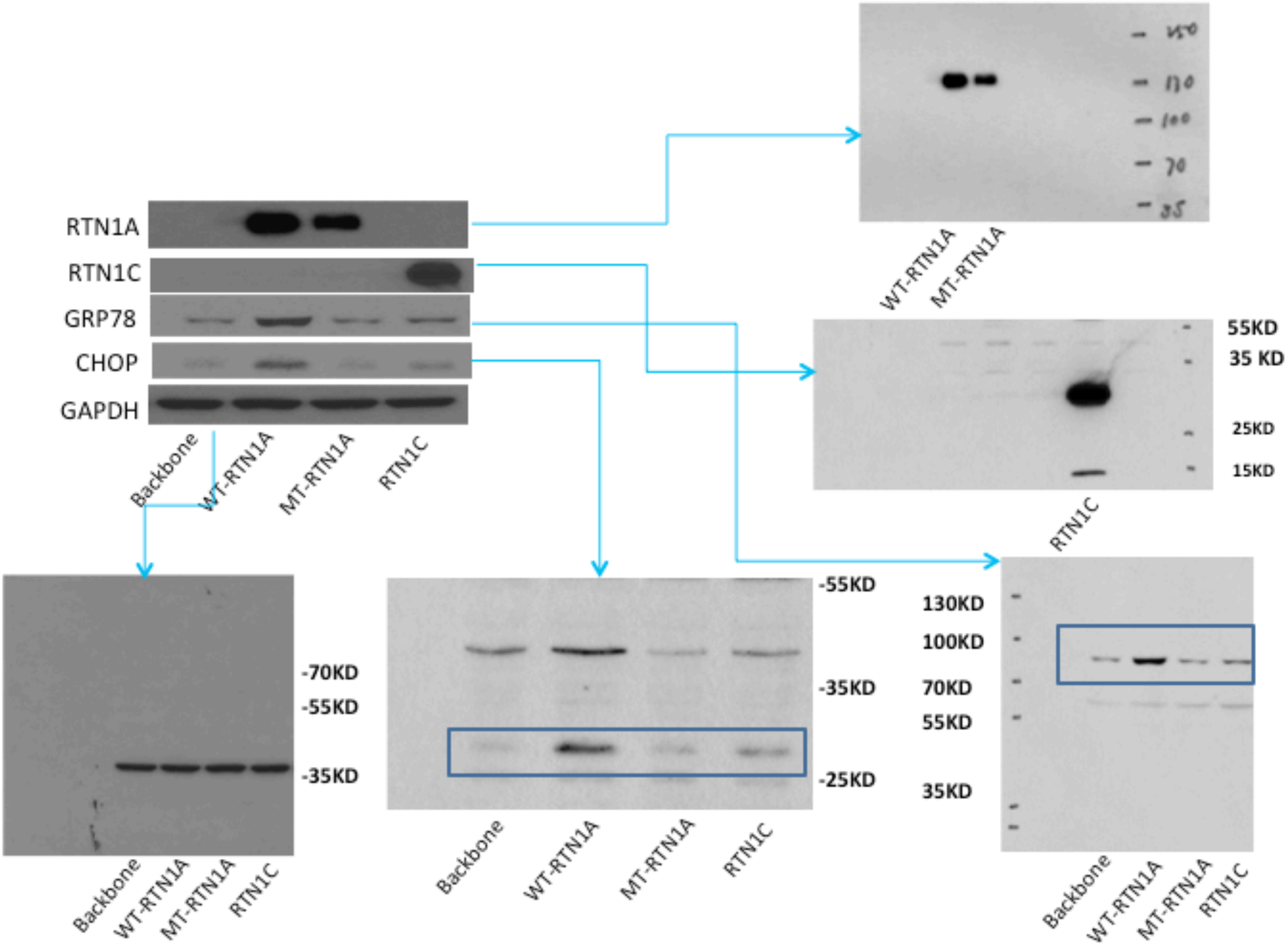

RTN1A
RTN1C
GRP78
CHOP
GAPDH
Backbone
WT-RTN1A
MT-RTN1A
RTN1C
WT-RTN1A
MT-RTN1A
55KD
35 KD
25KD
15KD
RTN1C
-70KD
-55KD
-35KD
Backbone
WT-RTN1A
MT-RTN1A
RTN1C
-55KD
-35KD
-25KD
Backbone
WT-RTN1A
MT-RTN1A
RTN1C
130KD
100KD
70KD
55KD
35KD
Backbone
WT-RTN1A
MT-RTN1A
RTN1C

Figure 7C

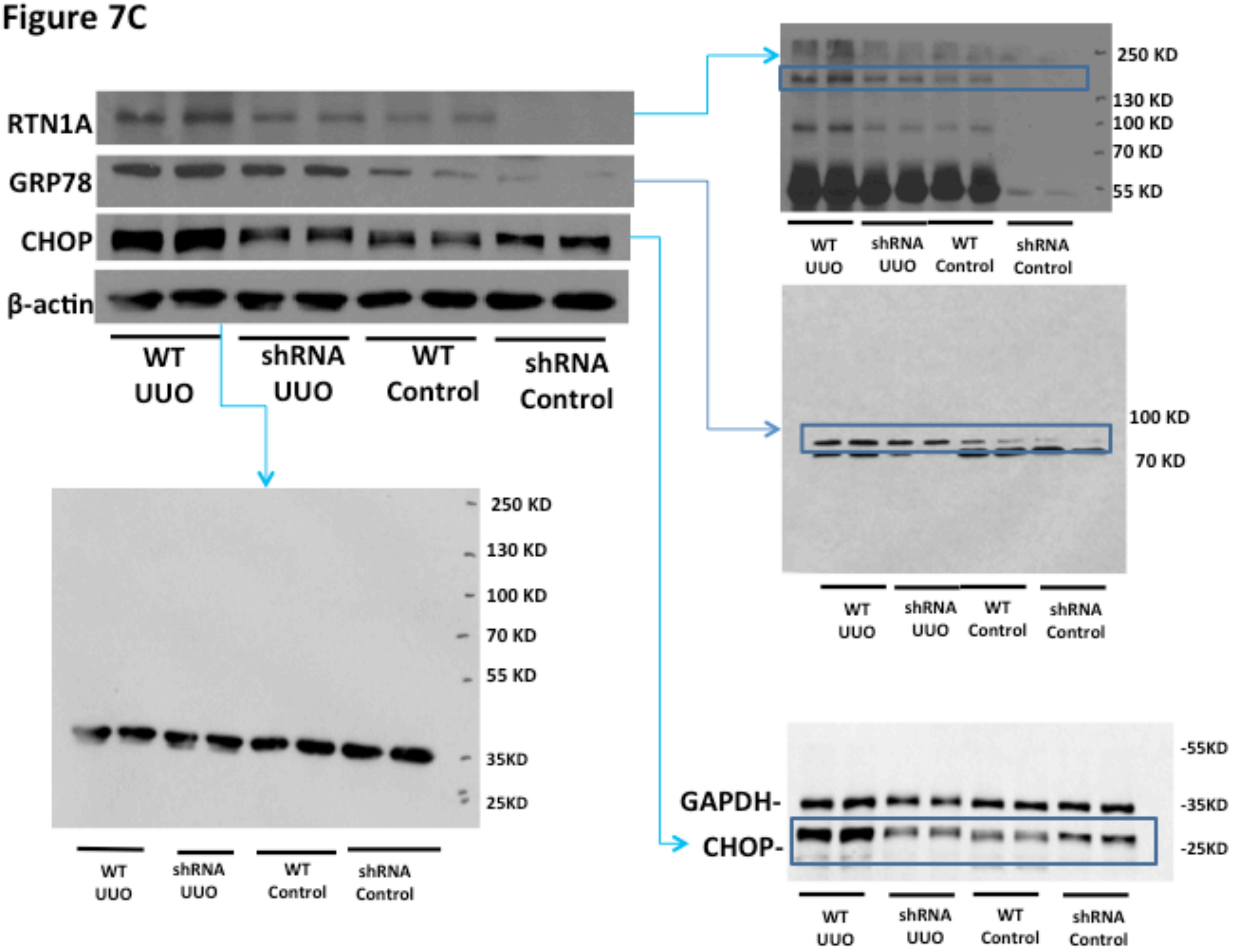

Supplementary Figure 20: Images of full-length western blots used for main figures.
